# A Multi-Strategy Improvement Secretary Bird Optimization Algorithm for Engineering Optimization Problems

**DOI:** 10.3390/biomimetics9080478

**Published:** 2024-08-08

**Authors:** Song Qin, Junling Liu, Xiaobo Bai, Gang Hu

**Affiliations:** 1 School of Art and Design, Xi’an University of Technology, Xi’an 710054, China; qinbinbin@126.com (S.Q.); xiaobo413@126.com (X.B.); 2 National Demonstration Center for Experimental Arts Education, Nankai University, Tianjin 300371, China; 3 Department of Applied Mathematics, Xi’an University of Technology, Xi’an 710054, China; hugang@xaut.edu.cn

**Keywords:** secretary bird optimization algorithm, feedback regulation mechanism, golden sinusoidal guidance, population diversity, engineering optimization, shape optimization model

## Abstract

Based on a meta-heuristic secretary bird optimization algorithm (SBOA), this paper develops a multi-strategy improvement secretary bird optimization algorithm (MISBOA) to further enhance the solving accuracy and convergence speed for engineering optimization problems. Firstly, a feedback regulation mechanism based on incremental PID control is used to update the whole population according to the output value. Then, in the hunting stage, a golden sinusoidal guidance strategy is employed to enhance the success rate of capture. Meanwhile, to keep the population diverse, a cooperative camouflage strategy and an update strategy based on cosine similarity are introduced into the escaping stage. Analyzing the results in solving the CEC2022 test suite, the MISBOA both get the best comprehensive performance when the dimensions are set as 10 and 20. Especially when the dimension is increased, the advantage of MISBOA is further expanded, which ranks first on 10 test functions, accounting for 83.33% of the total. It illustrates the introduction of improvement strategies that effectively enhance the searching accuracy and stability of MISBOA for various problems. For five real-world optimization problems, the MISBOA also has the best performance on the fitness values, indicating a stronger searching ability with higher accuracy and stability. Finally, when it is used to solve the shape optimization problem of the combined quartic generalized Ball interpolation (CQGBI) curve, the shape can be designed to be smoother according to the obtained parameters based on MISBOA to improve power generation efficiency.

## 1. Introduction

Optimization problems in engineering design are to select a set of parameters (variables) to achieve the optimal value of the design index (goal) under a series of relevant constraints [1]. Those confusions widely exist in various fields, such as production cost optimization [2], public transportation optimization [3], production decision optimization [4], structural design optimization [5], feature selection [6], path planning [7], and so on.

To solve those complex optimization problems effectively, meta-heuristic algorithms (MHAs) are studied and proposed, the core idea of which is to gradually approach the optimal solution of the problem by searching in the problem space [8]. Different from optimization algorithms based on precise gradient information or Hesse matrix, the main advantage of MHAs is that they can handle complex, non-linear problems and do not require assumptions about the specific model of the problem [9]. Furthermore, with the continuous development and improvement of computer hardware and software, MHAs have shown strong ability to solve practical problems, and the future development prospects will be broader [10].

**Figure 1:**
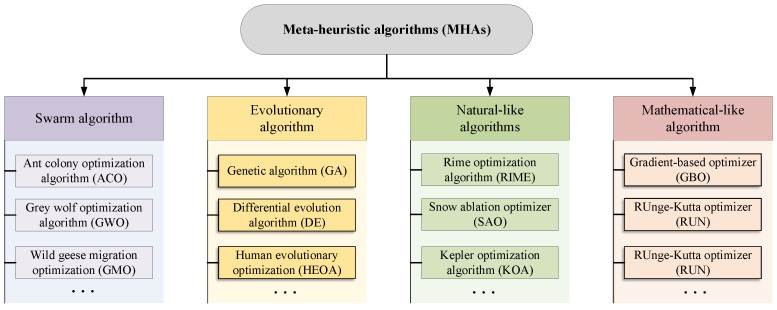
The classification of MHAs.

As shown in Figure 1, according to different search mechanisms, MHAs are usually classified into four types, which are swarm algorithms, evolutionary algorithms, natural-like algorithms, and mathematical-like algorithms [11]. For swarm algorithms, they currently are the most popular MHAs, inspired by the adaptive, self-organizing group behaviors exhibited by social species in nature, such as foraging, hunting, working, or migrating [12]. For example, in the classical ant colony optimization algorithm (ACO), the foraging behavior based on pheromone concentration in the ant group is modeled mathematically to form a basic search framework [13]. Grey wolf optimization algorithm (GWO) was constructed by simulating the hunting behavior of grey wolves, belonging to swarm algorithms. Based on the hierarchical mechanism and hunting behavior, the special mechanism has achieved good results in balanced exploration and development of GWO, leading to good performance in convergence speed and solution accuracy [14]. Inspired by the migratory behavior of wild geese, a wild geese migration optimization (GMO) algorithm was developed. During the long-distance migration, they usually keep a special formation consisting of several small groups [15]. Evolutionary algorithms are constructed by the natural law of survival of the fittest [16]. For genetic algorithm (GA), it simulated theory of Darwinian biological evolution, mainly including natural selection and genetic mechanisms [17]. Differential evolution algorithm (DE) was also a representative of this type. It is inspired by the mutation strategy, hybridization strategy, and selection operations [18]. Different from GA, each individual in the DE corresponds to a solution vector, and the complex encoding and decoding process is abandoned. Human evolutionary optimization algorithm (HEOA) was inspired by the human evolution process, mainly consisting of two distinct phases: human exploration and human development [19]. Natural-like algorithms are developed by modeling common natural phenomena, such as rain, snow, wind, and so on [20]. For example, inspired by the mechanism of frost and ice growth in nature, a novel rime optimization algorithm (RIME) was proposed in 2023. By studying the various growth processes of rime-ice, this algorithm develops a novel searching frame, including soft-rime search and hard-rime puncture strategies [21]. A snow ablation optimizer (SAO) was constructed in 2022, which mainly emulates the sublimation and melting behavior of snow to realize a balance between exploitation and exploration in the solution space and discourage premature convergence [22]. The Kepler optimization algorithm (KOA) was also a well-known approach of natural-like algorithms based on the celestial motion principle, which simulates the motion law of planets around the sun to solve the optimization problem [23]. The last category is mathematical-like algorithms, mainly including the sine cosine algorithm (SCA) [24], gradient-based optimizer (GBO) [25], and Runge Kutta optimizer (RUN) [26]. The SCA only uses the volatility and periodicity of sine and cosine functions as the design goal of the operator to search and iterate the optimal solution. Compared with the GA, SCA has the advantages of fewer parameters, a simple structure, and easy implementation. For the GBO, it adopts a gradient-based approach to improve exploration trends and accelerate convergence to obtain better bits in the search space.

Though optimization algorithms can be solved by various MHAs, the accuracy and efficiency are different due to diverse algorithm structures. Thus, to enhance the performance of original algorithms, different improved versions of the basic MHAs are introduced [27]. Here, two common types of improvement methods are discussed. In the first class, hybrid algorithms are constructed by mixing different basic algorithms with various characters [28]. For example, Nenavath and Jatoth proposed a new hybrid SCA-DE by mixing the SCA and differential DE [29]. Compared with the basic SCA and DE, the proposed hybrid algorithm has better capability to escape from local optima with faster convergence. Garg also presented a hybrid algorithm named PSO-GA for solving constrained optimization problems. It adopted the direction of particle swarm optimization (PSO) and the decision operators of GA, leading to better balance between the exploration and exploitation abilities [30]. However, this kind of method may inherit their own absence when combining the advantages of different algorithms, which means certain limitations on the improvement effect. Thus, another kind of method is studied, that is, several improvement strategies are incorporated into the original algorithms. For example, aiming to overcome the shortcomings of low accuracy, slow convergence speed, and easily falling into local optimums, Hu et al. proposed an improved BWO algorithm by introducing novel selecting strategies, mutation methods, and adaptive parameters [31]. The proposed algorithm achieved higher classification accuracy while using fewer information from the original datasets. Liu et al. proposed an enhanced grey wolf optimization algorithm (NAS-GWO) for the agricultural UAV trajectory planning problem [32]. The improved algorithm mainly includes three key strategies. A boundary constraint mechanism is adopted to enhance the ability of achieving better solutions. The typical Gaussian mutation model and spiral function are used to reduce the possibility of being troubled with local optimums. Meanwhile, to keep the balance of exploitation and exploration abilities, the NAS-GWO hires a nonlinear factor based on the Sigmoid function.

In this paper, a novel secretary bird optimization algorithm (SBOA) proposed in 2024, is analyzed and studied. By being tested on CEC2017 and CEC2022 benchmark test suites, the searching accuracy, convergence rate, and stability of SBOA have been verified [33]. However, limited by the single structure of the original algorithm, there is still room to develop an enhanced version to get better solutions. Thus, by introducing targeted improvement strategies, we propose a multi-strategy secretary bird optimization algorithm (MISBOA). The main contributions can be summarized as follows:

(1) An enhanced algorithm MISBOA is proposed by integrating four specific improvement strategies.
  A feedback regulation mechanism is introduced to fully make use of the global information, leading to faster global convergence.
  To improve the development ability of SBOA, a golden sinusoidal guidance strategy is adopted in the hunting stage.
  In the escape stage, a cooperative camouflage strategy is employed to enhance the global exploration ability.
  An update strategy based on cosine similarity is used to keep the population diverse, which influences the ability to escape local optimums.
(2) The ability of MISBOA is verified by solving basic test functions and well-known engineering optimization problems.
  The MISBOA and other eight typical algorithms are tested on functions with 10 and 20 dimensions of CEC2022. Accuracy, convergence speed, stability, and extensibility of various algorithms are analyzed.
  The engineering application capability of the MISBOA is verified on five complex engineering optimization problems.
(3) The MISBOA is applied for a shape optimization problem of combined curves.
  With the aim of minimizing the total energy, a shape optimization model of combined quartic generalized Ball interpolation (CQGBI) curve is established.
  The shape of wind-driven generator blades is optimized by MISBOA and other algorithms to increase the efficiency of power generation.

**Figure 2:**
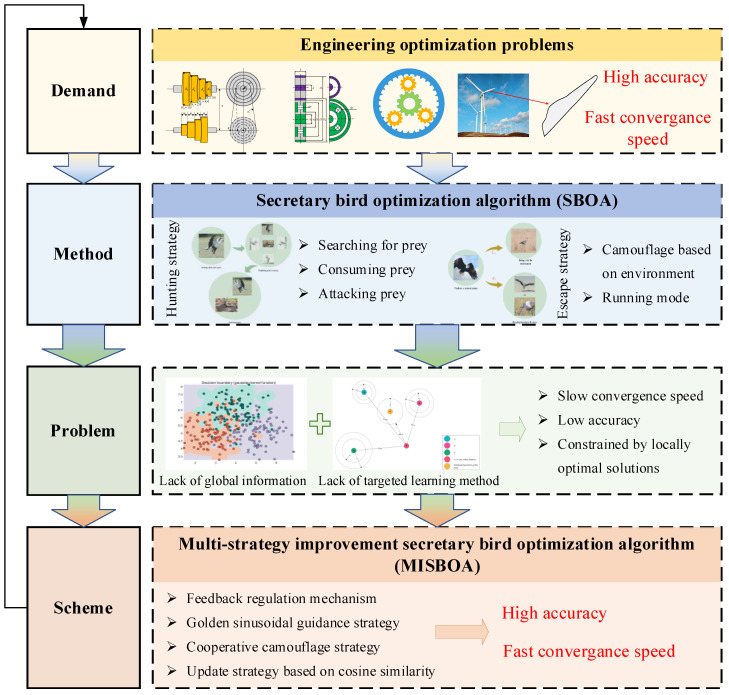
The motivation of this paper.

Figure 2 shows the motivation of this paper to illustrate the idea and value of the research more intuitively. The organization of our work can be summarized as follows: Section 2 introduces the original SBOA algorithm and the detailed steps for constructing the MISBOA. Section 3 analyzes the performance of MISBOA according to the results of the CEC2022 test suit. Section 4 tests the ability of MISBOA in solving engineering optimization problems and the established shape optimization model of combined quartic generalized Ball interpolation. In Section 5, the main conclusions of this paper and future work are given.

## 2. The Multi-Strategy Improvement Secretary Bird Optimization Algorithm

### 2.1. The Basic Secretary Bird Optimization Algorithm

Secretary birds are large terrestrial raptors, usually inhabit tropical savannas or semi-desert areas. They are natural enemies of snakes on the African continent, such as the black mamba, cobra, and others. Inspired by their survival strategy of searching for prey and evading pursuit from predators in harsh environments, a secretary bird optimization algorithm is constructed to deal with complex optimization problems. The SBOA consists of the following parts:

#### 2.1.1. Initial Preparation Phase

Firstly, for a typical minimization optimization problem f(x), the initial solutions used to start the search needs to be determined. Here, *N* initial solutions form the initial population $X=[X_{1},X_{2},\cdots ,X_{N}]$ of secretary birds. Each individual *X_i_* in the population corresponds to a solution to the optimization problem, which is initialized by Equation (1).

(1) $$X_{i}=lb+rand\cdot (ub-lb),\;\;\;\;1\leq i\leq N,$$

where *lb* and *ub* are the lower and upper bounds of the decision variables. *rand* represents a random number in [0, 1]. *M* is the dimension of the problem. In addition, the fitness value of the solution *X_i_* is noted as $F_{i}=f(X_{i})$ to measure its quality.

#### 2.1.2. Hunting Strategy of Secretary Birds

Unlike other fierce predators, secretary birds adopt a more intelligent approach to hunting snakes. After spotting the target, they do not dive down and fight straightaway, but wander, jump, and pick quarrels near the snake patiently to observe and confuse opponents. Until a suitable time, they will strike quickly and kill the prey. Thus, the whole hunting process can be divided into three steps, which are searching for prey, consuming prey, and attacking prey. Then, mathematical models are used to characterize these stages.

(1) Searching for prey

**Figure 3:**
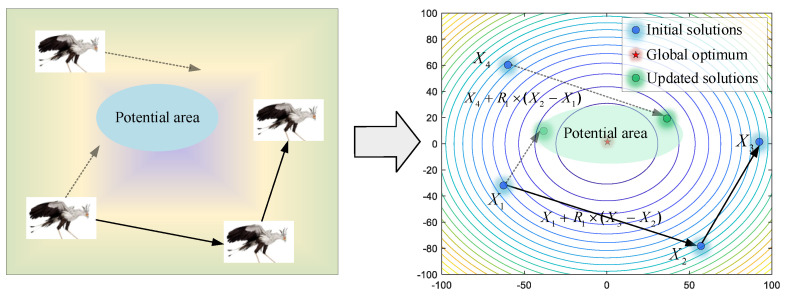
The modeling process of searching for prey.

In this stage, the secretary birds need to search for prey at a safe distance. For optimization algorithms, this initial stage asks for stronger exploration ability to acquire enough information for the whole search area. As shown in Figure 3, the secretary bird can explore new potential areas by referring to the positions of the other two secretary birds. Therefore, differential mutation operations are introduced to keep enhancing algorithm diversity. For each individual *X_i_*, it will use Equations (2) and (3) to update position when the current iteration time *t* is smaller than one-third of the maximum iterations *T*.

(2) $$X_{i}^{new}(t)=X_{i}(t)+(X_{r_{1}}(t)-X_{r_{2}}(t))\times R_{1}$$

(3) $$\left\{\begin{array}{ll} X_{i}(t+1)=X_{i}^{new}(t), & \;\mathrm{if}\;F_{i}^{new}<F_{i}\\ X_{i}(t+1)=X_{i}(t), & \;\mathrm{else} \end{array}\right.$$

where $X_{r_{1}}(t)$, $X_{r_{2}}(t)$ are two individuals randomly selected from the current population. *R*_1_ is a random vector, including 1 × *M* elements randomly selected in [0, 1].

(2) Consuming prey

**Figure 4:**
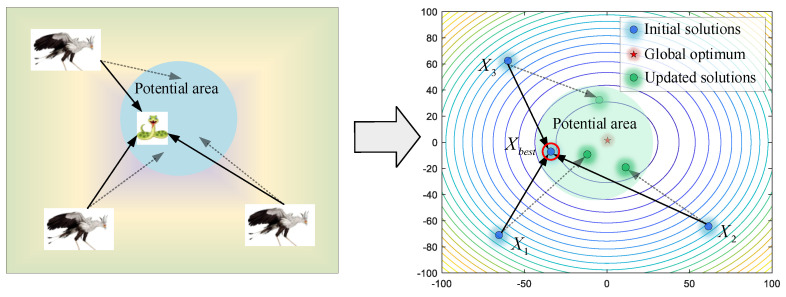
The modeling process of consuming prey.

Once secretary birds find potential prey, they first hover around the snake with agile footwork and maneuvers. By watching and luring opponents in the process of circling, the patience of the prey will be consumed to let down its guard. As shown in Figure 4, by regarding the current best individual as the prey, other secretary birds update their position to close the prey. In this way, the success of hunting will be greatly improved. When $\frac{1}{3}T<t<\frac{2}{3}T$, the Brownian motion (*RB*) is applied to model the random movement of the secretary birds, as shown in Equation (4),

(4) RB=randn(1,M)

where *randn*(1,*M*) is a randomly generated vector satisfying the standard normal distribution, whose mean value is 0 and standard deviation is 1.

Then, secretary birds update their positions by Equations (5) and (6).

(5) $$X_{i}^{new}(t)=X_{best}(t)+\exp({\left(t/T\right)}^{4})\times (RB-0.5)\times (X_{best}(t)-X_{i}(t))$$

(6) $$\left\{\begin{array}{ll} X_{i}(t+1)=X_{i}^{new}(t), & \;\mathrm{if}\;F_{i}^{new}<F_{i}\\ X_{i}(t+1)=X_{i}(t), & \;\mathrm{else} \end{array}\right.$$

where *X_best_* is the best solution for the current population.

(3) Attacking prey

**Figure 5:**
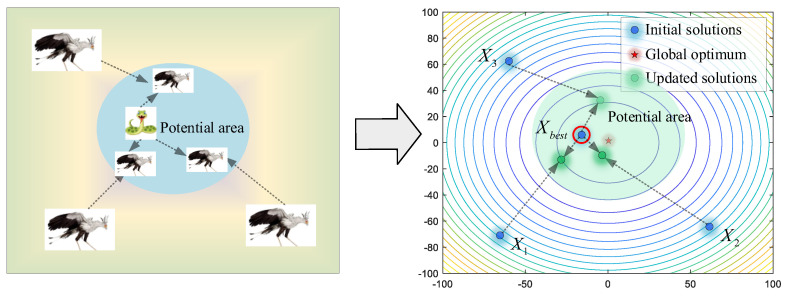
The modeling process of attacking prey.

After constant consumption, the prey will be exhausted. It is time for secretary birds to start the attack. Here, the levy flight strategy is used to simulate various attack ways, such as continuous steps and occasional long jumps in a short time. Equations (7) and (8) are used to describe the characters of this stage. As shown in Figure 5, secretary birds quickly approach the prey, meaning candidate solutions are close to the current best solution. When $t\geq \frac{2}{3}T$, this strategy will be executed.

(7) $$X_{i}^{new}(t)=X_{best}(t)+\left({\left(1-t/T\right)}^{\left(2\times t/T\right)}\right)\times X_{i}(t)\times RL$$

(8) $$\left\{\begin{array}{ll} X_{i}(t+1)=X_{i}^{new}(t), & \;\mathrm{if}\;F_{i}^{new}<F_{i}\\ X_{i}(t+1)=X_{i}(t), & \;\mathrm{else} \end{array}\right.$$

where *RL* represents the levy flight strategy, which is

(9) RL=0.5×Levy(M)

where $Levy(M)=s\times \frac{u\times \sigma }{{\left|v\right|}^{1/\varphi }}$, in which *s* and *φ* are fixed numbers of 0.01 and 1.5, respectively. *u* and *υ* are random numbers in [0, 1]. *σ* is set as follows.

(10) $$\sigma ={\left(\frac{\Gamma (1+\eta )\times \sin\left(\frac{\pi \eta }{2}\right)}{\Gamma \left(\frac{1+\eta }{2}\right)\times \eta \times 2\left(\frac{\eta -1}{2}\right)}\right)}^{1/n}$$

where $\Gamma \left(\cdot \right)$ is the gamma function and *η* is set as 0.5.

#### 2.1.3. Escape Strategy for Secretary Birds

In nature, secretary birds also face the risk of being hunted when hunting other prey. The main enemies they need to face are eagles, hawks, foxes, and jackals. When they sense danger, various evasion strategies are necessary to protect themselves or their food. In this algorithm, camouflage and running modes are modeled to simulate the escape strategies.

(1) Camouflage based on environment

**Figure 6:**
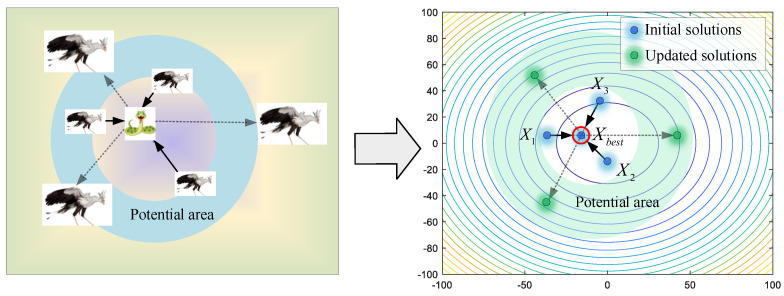
The modeling process of camouflage based on environment.

Facing enemies, the secretary birds first choose to camouflage themselves to avoid danger. As shown in Figure 6, secretary birds update their positions around the prey (best individual), reflecting the behavior of trying to escape local optimums in the algorithms. The mathematical model of this strategy is shown in Equations (11) and (12).

(11) $$X_{i}^{new}(t)=X_{best}(t)+(2\times RB-1)\times {\left(1-t/T\right)}^{2}\times X_{i}(t)$$

(12) $$\left\{\begin{array}{ll} X_{i}(t+1)=X_{i}^{new}(t), & \;\mathrm{if}\;F_{i}^{new}<F_{i}\\ X_{i}(t+1)=X_{i}(t), & \;\mathrm{else} \end{array}\right.$$

(2) Running mode

**Figure 7:**
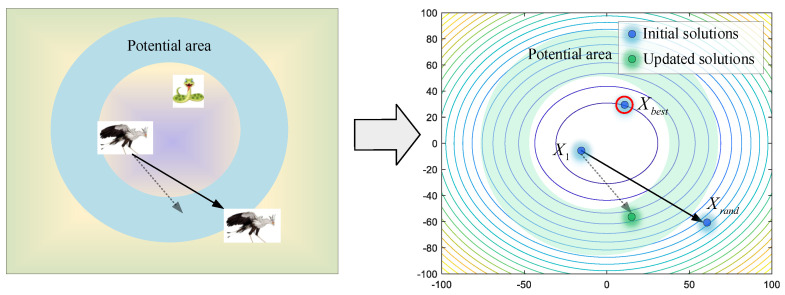
The modeling process of running mode.

If they cannot avoid the enemy, flight or rapid-running strategies will be employed to keep them safe. As shown in Figure 7, a random individual *X_rand_* is selected as the leader for reference, avoiding being limited to local optimums. Secretary birds update their positions by Equations (13) and (14).

(13) $$X_{i}^{new}(t)=X_{best}(t)+R_{2}\times (X_{rand}(t)-K\times X_{i}(t))$$

(14) $$\left\{\begin{array}{ll} X_{i}(t+1)=X_{i}^{new}(t), & \;\mathrm{if}\;F_{i}^{new}<F_{i}\\ X_{i}(t+1)=X_{i}(t), & \;\mathrm{else} \end{array}\right.$$

**Figure 8:**
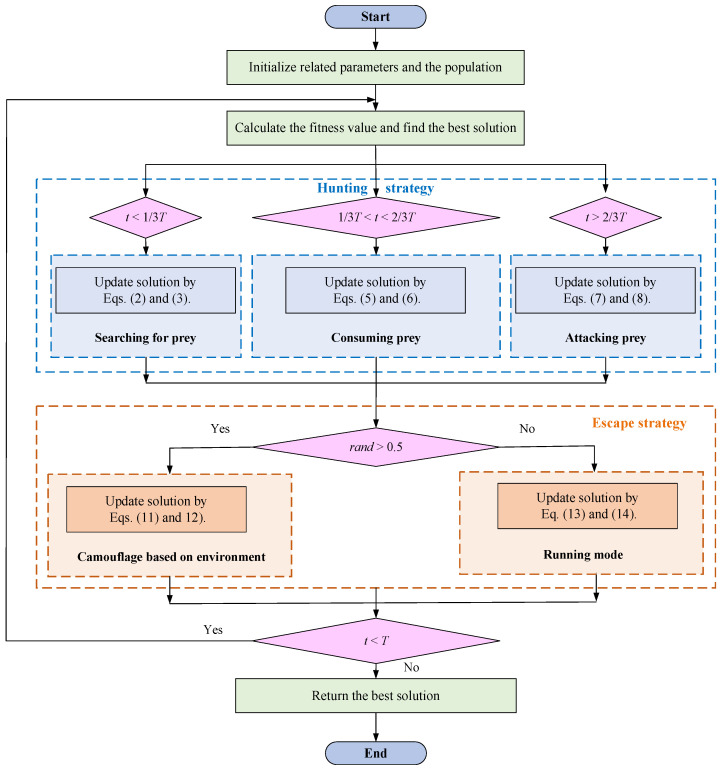
The flowchart of the SBOA algorithm.

The above modes will be executed with equal probability to show the randomness of searching for the feasible area. In addition Figure 8 shows the flow chart of the original SBOA algorithm.

### 2.2. The Multi-Strategy Improvement Secretary Bird Optimization Algorithm

Though the SBOA has shown excellent performance in solving various test functions compared with the other 15 advanced algorithms, there are still some shortcomings in the algorithm construction, which can be further improved to obtain faster convergence and solutions with higher accuracy. The main inadequacy is reflected in the following four aspects.

(1) For the whole algorithm, the global information is not fully used to adjust the position update strategy. This defect may affect the overall performance of the SBOA, which means the balance between development ability and exploration ability.
(2) In the hunting stage, the attack mode of secretary birds can be further enhanced by observing the performance of the prey, which is to improve the development ability of SBOA.
(3) In the escape stage, secretary birds decide how to camouflage or escape using a few simple random strategies, which may influence the ability to escape local optimums.

Aiming at the above problems, this section will introduce the appropriate strategy to construct a novel version of SBOA.

#### 2.2.1. Feedback Regulation Mechanism

**Figure 9:**
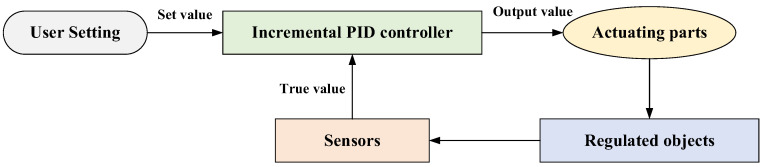
Schematic diagram of the incremental PID control.

Feedback regulation mechanism is a common structural design in the biological world. It can regulate the state of the system at the next stage according to the result at the current stage after receiving the stimulus of internal and external environmental changes (Figure 9). In the field of engineering control, the PID controller is one of the most widely used automatic controllers, which is constructed by the idea of feedback regulation [34]. Among various PID methods, incremental PID control is a typical recursive algorithm, which makes the system stable by adjusting the controlled object according to the output value [35]. This kind of control method can be used in the optimization algorithm, by regarding the searching process as a system and the best fitness value in each iteration as the output value. Thus, the discrete incremental PID control is adopted to form the feedback regulation mechanism.

Firstly, the system deviations need to be defined as Equation (15).

(15) $$e_{k}(t)=X_{best}(t)-X(t)$$

where $X_{best}(t)$ is the best solution in the *t*th iteration. To facilitate the following calculation, the deviation of the previous iteration is denoted as $e_{k-1}(t)$. The deviation of the previous two iterations is denoted as $e_{k-2}(t)$. When *t* = 1, let $e_{k-2}(t)=e_{k-1}(t)=e_{k}(t)$. When *t >* 1, $e_{k-2}(t)=e_{k-1}(t-1)$. Furthermore, to reduce the space complexity, the $e_{k-1}(t)$ can be calculated by

(16) $$e_{k-1}(t)=e_{k}(t-1)+X_{best}(t)-X_{best}(t-1)$$

Then, the output of PID regulation in iteration *t* is

(17) $$\Delta u=K_{p}\cdot r_{1}\cdot (e_{k}(t)-e_{k-1}(t))+K_{i}\cdot r_{2}\cdot e_{k}(t)+K_{d}\cdot r_{3}\cdot (e_{k}(t)-2e_{k-1}(t)+e_{k-2}(t))$$

where *r*_1_, *r*_2_, *r*_3_ are random numbers from 0 to 1. *K_p_*, *K_i_*, and *K_d_* are adjustment coefficients for the proportion, integral and differential, respectively.

Finally, the updated solutions can be calculated by the following equation:

(18) X(t+1)=X(t)+λ⋅Δu(t)+(1−λ)⋅H(t)

where $\lambda =r_{4}\cos(t/T)$. $H(t)=(\cos(1-t/T)+\rho \cdot r_{5}\cdot L)\cdot e_{k}(t)$ is a conditioning factor to prevent the algorithm from falling into local optimum, in which $\rho ={\left(\ln(T-t+2)/\ln(T)\right)}^{2}$ and *L* is a *Levy* flight function. And *r*_4_ and *r*_5_ are random numbers from 0 to 1.

#### 2.2.2. Golden Sinusoidal Guidance Strategy

After locking the prey, the secretary bird will start to attack the target. To enhance the success rate of capture, this section will adopt a golden sinusoidal guidance strategy to take advantage of the target’s location information [36]. In the algorithm, this strategy is useful to enhance the ability of developing local areas. For the individual *i* in the population, it will update its position by Equation (19).

(19) $$X_{i}(t+1)=X_{i}(t)\times \left|\sin(s_{1})\right|+s_{2}\times \left|\sin(s_{1})\right|\times \left|\theta_{1}\times X_{best}(t)-\theta_{2}\times X_{best}(t)\right|$$

where *s*_1_ is a random value in [0, 2π], and *s*_2_ is a random value in [0, π]. *θ*_1_ and *θ*_2_ are the coefficients obtained by the golden section, which are $\theta_{1}=-\pi +2\pi (1-\tau )$ and $\theta_{1}=-\pi +2\pi \tau$, in which τ is defined as $\tau =\frac{1-\sqrt{5}}{2}$.

#### 2.2.3. Cooperative Camouflage Strategy

When secretary birds feel threatened, they will first use the environment to disguise themselves. However, this kind of camouflage strategy cannot effectively avoid enemy attacks because the information exchange between populations is neglected in the original method. Thus, this section will introduce a cooperative camouflage strategy by considering the positions of different individuals. First, three different individuals need to be selected, noted as *X_a_*, *X_b_*, and *X_c_*. A new solution just can be generated by Equation (20).

(20) $$X_{i}(t+1)=X_{a}+r_{6}\times (X_{b}-X_{c})$$

where *r*_6_ is a random number from 0 to 1.

#### 2.2.4. Update Strategy Based on Cosine Similarity

When the camouflage strategy does not work, secretary birds have to run to escape enemies. Then, the key to success is how to choose the right escape direction. Generally speaking, an empty place will be the first choice to ensure sufficient space. For the individual *X_i_*, the cosine similarity is used to measure the crowding degree to other individuals.

Firstly, construct vectors *A* and *B* by Equation (21).

(21) $$\left\{\begin{array}{l} A=X_{i}(t)-X_{best}(t)\\ B=X_{i}(t)-X_{best}(t) \end{array}\right.$$

where *i* ≠ *j*.

Then, calculate the similarity between *X_i_* and *X_j_* by Equation (22).

(22) $$\psi_{i,j}=\frac{A\cdot B}{\left|A\right|\left|B\right|}$$

After comparing *X_i_* and others, the individual with the smallest cosine similarity will be selected as the update direction, noted as *X_s_*. A new solution can be obtained by Equation (23).

(23) $$X_{i}(t+1)=X_{best}(t)+R\times (X_{s}(t)-K\times X_{i}(t))$$

where *R* is a vector including random elements in [0, 1], and *K* is an integer, randomly toggling between 1 and 2.

**Figure 10:**
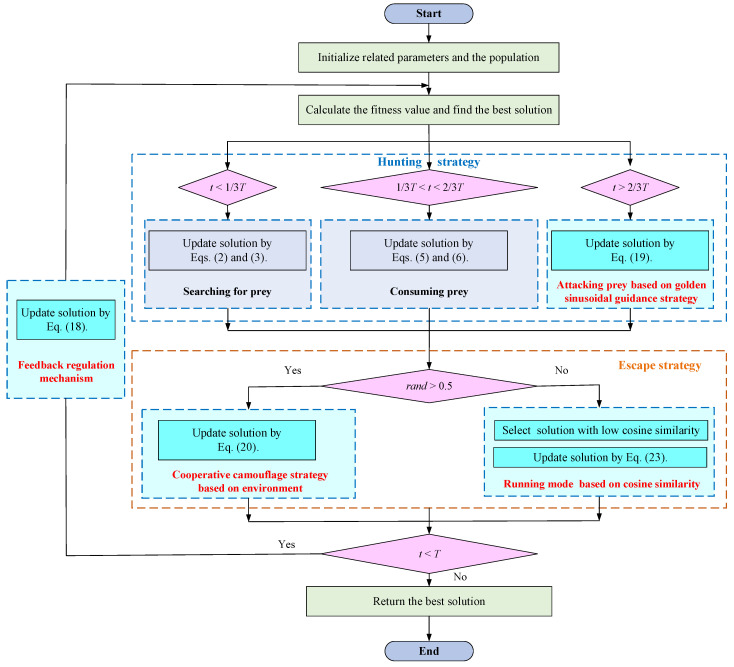
The flowchart of the MISBOA algorithm.

Figure 10 shows the flowchart of the MISBOA algorithm. The specific steps of the MISBOA algorithm are as follows:

Step 1. Initialize the related parameters and the population of secretary birds.

Step 2. Calculate the fitness value of each solution and obtain the best solution in the current population.

Step 3. According to the historical information, update solutions based on the feedback regulation mechanism in Equation (18).

Step 4. If *t* < *T*/3, secretary birds search for the prey in the hunting stage and update solutions by Equations (2) and (3).

Step 5. If *T*/3 < *t* < 2*T*/3, secretary birds start to consume the prey in the hunting stage and update solutions by Equations (5) and (6).

Step 6. If *t* > 2*T*/3, secretary birds are attacking the prey based on the Golden sinusoidal guidance strategy in the hunting stage and update solutions by Equation (19).

Step 7. Turn to the escape stage. If *rand* > 0.5, update solutions by Equation (20) based on the cooperative camouflage strategy; otherwise, update solutions by Equation (23) based on the cosine similarity.

Step 8. If *t* < *T*, turn to Step 1; otherwise, output the best solution.

#### 2.2.5. Computational Complexity Analysis

Computational complexity is a significant metric to measure whether an optimization algorithm can be deployed and applied quickly. Based on the Big O notation, the computational complexity of basic SBOA is *O*(*N* × *T* × (*Dim* + 1)), where *N*, *T*, *Dim* are the population size, maximum iterations, and dimensions, respectively. For the proposed MISBOA, four introduced strategies may potentially increase the computational complexity. However, the golden sinusoidal guidance strategy, cooperative camouflage strategy, and update strategy based on cosine similarity are more effective upgraded versions of the original methods, which do not incur additional computational costs. Thus, the computational complexity is *O*(*T* × *N*) + *O*(*T* × *N* × *Dim*) + *O*(*T* × *N* × *Dim*), resulting in *O*(*N* × *T* × (2 × *Dim* + 1)).

## 3. Numerical Experiment on the Test Functions

This section will test the comprehensive ability of the proposed MISBOA algorithm. By analyzing the results of MISBOA and other selected algorithms in solving test functions of the CEC2022 test suite, the effectiveness of the introduced strategy will be discussed.

### 3.1. Test Functions and Parameter Setting

For the objectivity and rationality of the experiment, it is inevitable to select other typical intelligent optimization algorithms as the control group. In this paper, other 8 popular algorithms are selected, which are the original SBOA [33], the golden jackal optimization algorithm (GJO) [37], the particle swarm optimization algorithm (PSO) [38], the PID-based search algorithm (PSA) [35], the quadratic interpolation optimization algorithm (QIO) [39], the Newton-Raphson-based optimizer (NRBO) [40], and the sand cat swarm optimization algorithm (SCSO) [41]. The parameters of the other metaheuristic algorithms are listed in Table 1.

For the CEC2022 test suite, there are 12 test functions, including unimodal functions, basic functions, hybrid functions, and composition functions. The dimension of test functions can be set as 10 or 20 [42]. Thus, for comprehensiveness and reliability, this section will test MISBOA and other comparison algorithms on the 12 test functions of 10 and 20 dimensions.

For the experiment conditions, the size of the initial population for all algorithms is set to 100, and the maximum iteration time is set to 1000. Then, the results of various algorithms will be recorded after 30 independent runs to avoid the influence of chance factors. The average value (Ave) and the standard deviation of the obtained 30 results will be used as the main indexes to evaluate the quality of various algorithms. Another index rank is determined by arranging the average values in ascending order. The smaller ranking result represents better performance. Average time cost (in seconds) for each function after 30 runs is also recorded to reflect the computational complexity of various algorithms.

### 3.2. Analysis and Discussion of the Results on CEC 2022 with 10 Dimensions

Firstly, the solving results of CEC 2022 with 10 dimensions are summarized in Table 2. The data marked in bold means the best results among all algorithms for the according evaluation index. Results show that the constructed MISBOA algorithm has the best performance for 6 test functions, followed by SBOA and QIO, which perform best on 1 and 4 test functions. Comparing the MISBOA and the basic SBOA, MISBOA can keep advantage over SBOA on 10 test functions, except for CEC09 and CEC12. For CEC03 and CEC05, the MISBOA can achieve the theoretical optimums after each run. However, the SBOA can only obtain the ideal results in a few experiments, which leads to a larger standard deviation. For the QIO algorithm, it can also rank first on 4 test functions, but its performance is not stable for all functions with various characters. For example, QIO only ranks fourth on CEC01, CEC02, CEC03, and CEC08 and ranks eighth on CEC12. The improved MISBOA ranks in the top three on all functions. It illustrates the introduction of multiple strategies effectively balances the capacities of exploration and development, further having stronger searching ability for various optimization problems. Based on the final ranking results, the performances of MISBOA and other algorithms are ranked as MISBOA > SBOA > QIO > PSA > PSO > PSOBKA > NRBO > SCSO > GJO. For time cost, the MISBOA inevitably consumes slightly more time compared to the original SBOA, which is consistent with the results of the complexity analysis. However, facing the NRBO and SCSO, the MISBOA emerges as the winner and competes evenly with the QIO algorithm. That is, the proposed MISBOA achieves higher solving accuracy while simultaneously minimizing increases in time costs as much as possible.

To support the above conclusions from statistical knowledge, Table 3 lists the *p*-value of the Wilcoxon rank sum test (WRST) between the proposed MISBOA and other algorithms. If the MISOBA performs better than another algorithm and the *p*-value is less than 0.05, it can illustrate the MISOBA is obviously superior to the comparison algorithm, noted ‘+’. Otherwise, if MISOBA performs worse than another algorithm and the *p*-value is less than 0.05, it is noted as ‘−’. If the *p*-value is over 0.05, there is no statistically significant difference between the two algorithms. From Table 3, compared with the SBOA, the improved algorithm gets the statistical advantage on 9 test functions and is obviously inferior to the original version on CEC12. For QIO, the MISBOA performs greatly better on 7 test functions and worse on 4 test functions. For BKA, GJO, PSO, NRBO, and SCSO, the advantages of all test functions of MISBOA can be supported by WRST.

**Figure 11:**
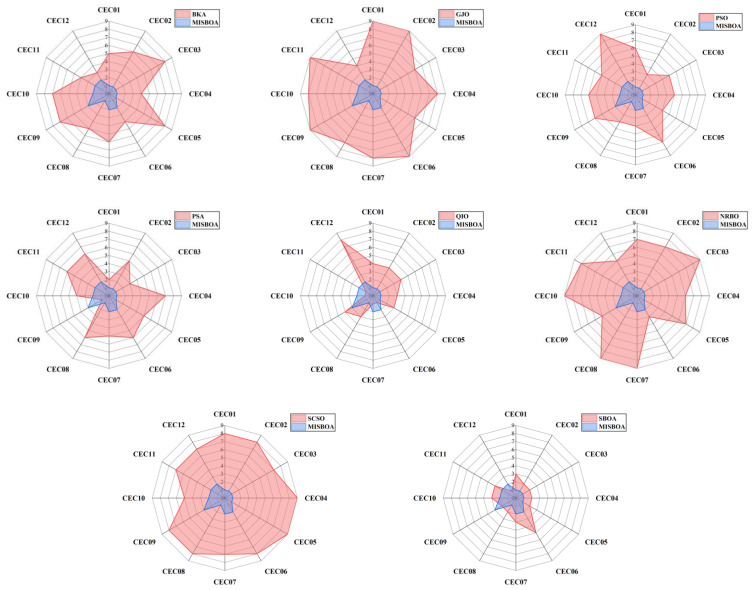
The radar comparison maps between MISBOA and other algorithms on CEC2022 with 10 dimensions.

**Figure 12:**
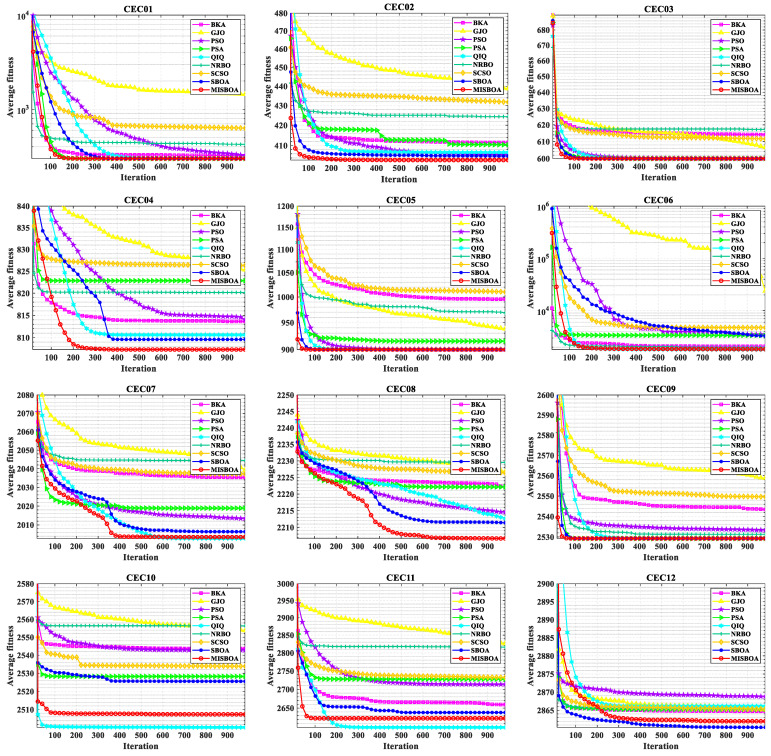
The average iterative curves of different algorithms on CEC2022 with 10 dimensions.

Figure 11 plots radar comparison maps between MISBOA and other algorithms on CEC2022 with 10 dimensions. A smaller radar coverage area means the algorithm has a more stable solving ability for various test functions in CEC2022. Compared with BKA, GJO, PSO, NRBO, and SCSO, the MISBOA takes advantage of all functions. For PSA, QIO, and SBOA, though the MISBOA cannot rank first on a few functions, its comprehensive performance is still excellent. The average iterative curves of different algorithms after 30 runs are shown in Figure 12. From the curves, convergence rate and accuracy can be visually observed and analyzed. Compared with the original SBOA, the convergence rate of the proposed algorithm is obviously faster than SBOA on CEC01, CEC02, CEC04, CEC06, CEC07, CEC08, CEC10, and CEC11 during the whole searching process. This kind of advantage comes from the comprehensive impact of multiple strategies. The introduction of the feedback regulation mechanism can constantly adjust updated strategies based on historical information to ensure the quality of the entire population. The golden sinusoidal guidance strategy also improves the local development capacity, leading to individuals converging to better solutions with faster speed. Thus, at the early stage, the curves of MISBOA fall faster. At the later stage, the cooperative camouflage strategy and update strategy based on cosine similarity play roles in improving the ability to keep the population diversity and avoiding the local optimums. Compared with the QIO, though the MISBOA is at a disadvantage on CEC10 and CEC11, the performance of the QIO on other test functions lacks stability.

### 3.3. Analysis and Discussion of the Results on CEC 2022 with 20 Dimensions

Then, to verify the scalability of the MISBOA algorithm, this section compares it with other selected algorithms on test functions in CEC2022 with 20 dimensions. With the increase of decision variables, the fast convergence ability of algorithms in the feasible space is further required. After 30 runs, the data is collected in Table 4. Except for CEC 04 and CEC10, the MISBOA ranks first on the rest 10 test functions. Compared with the results on CEC2022 functions with 20 dimensions, the advantages of MISBOA are further expanded. It illustrates the performance of MISBOA is still stable with the increase of dimensions. For the original SBOA, facing more decision variables, it ranks first on only 1 test function. That is, without the improved strategies, the basic algorithm cannot effectively search for solutions with higher accuracy. The performance of the QIO algorithm is similar, ranking first only on CEC10. Thus, the application of feedback regulation mechanisms, golden sinusoidal guidance strategies, cooperative camouflage strategies, and update strategies based on cosine similarity is a helpful approach to balancing the abilities of exploration and development. With increased dimensions, all algorithms incur higher time costs. However, the changes of MISBOA and SBOA are minimal compared to QIO, NRBO, and SCSO. That is, the structure of the MISBOA algorithm effectively mitigates the increase in time costs caused by growth in problem dimensions.

**Figure 13:**
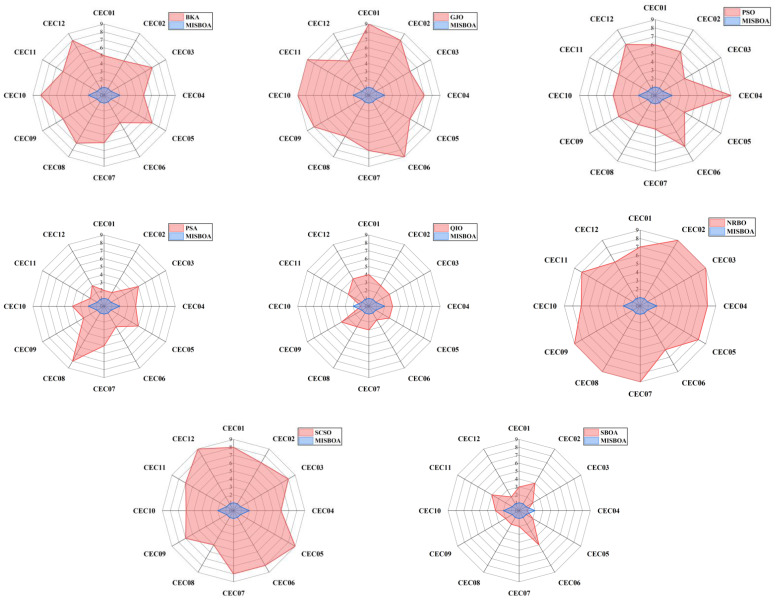
The radar comparison maps between MISBOA and other algorithms on CEC2022 with 20 dimensions.

Table 5 includes the *p*-value of WRST between MISBOA and other algorithms on CEC2022 with 20 dimensions. With the increase in dimensions, the difference between MISBOA and other algorithms is further revealed. For example, compared with the SBOA, MISBOA performs significantly better on 10 test functions under the conditions of 20 dimensions, more than that of 10 dimensions. This phenomenon is even more obvious when the MISBOA is compared with the QIO. The results of WRST prove that the MISBOA is obviously superior to QIO on 9 test functions and performs worse on only 1 test function. This result illustrates that the MISBOA is also effective when the dimension of problems is increased, while the performance of QIO is greatly affected. The radar comparison maps between MISBOA and others on CEC2022 with 20 dimensions in Figure 13 also prove that conclusion.

**Figure 14:**
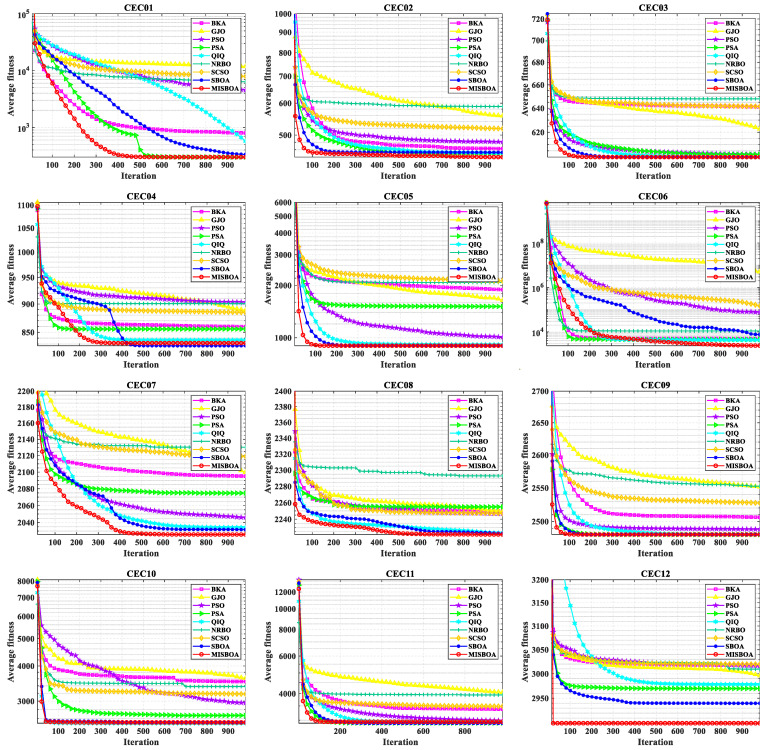
The average iterative curves of different algorithms on CEC2022 with 20 dimensions.

**Figure 15:**
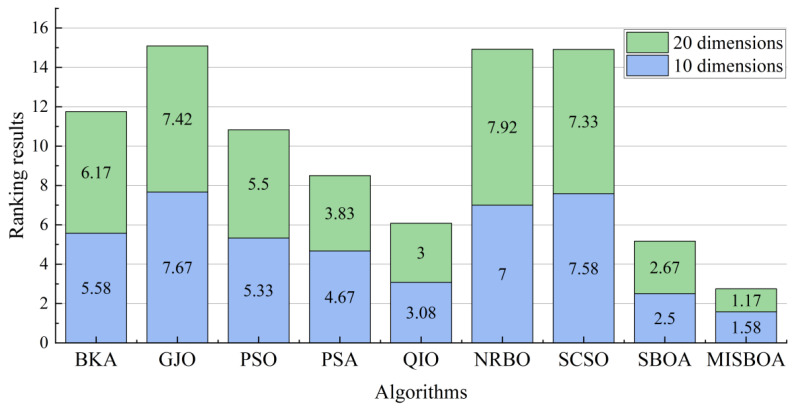
The statistical results of ranking on CEC2022 with 10 and 20 dimensions.

Figure 14 plots the average iterative curves of different algorithms on CEC2022 with 20 dimensions. It can be observed that the advantage of MISBOA in convergence speed is established in the beginning for all algorithms except for CEC06. It illustrates the value of the feedback regulation mechanism and golden sinusoidal guidance strategy. By referring to the historical information and the best solution in current, the whole population can quickly be close to better solutions. Meanwhile, for CEC06, when other algorithms cannot continue to find better solutions in the later stage, the iterative curve of MISBOA is still falling to explore feasible solutions with higher precision. It is the value of cooperative camouflage strategy and update strategy based on cosine similarity. By using the information of random individuals and individuals with small cosine similarity, the diversity of the population can be maintained to avoid local optimums. Figure 15 is the statistical results of ranking on CEC2022 with 10 and 20 dimensions. For MISBOA, it has the smallest ranking result for test functions with 10 and 20 dimensions, respectively. With the increase of decision variables, the average rank of MISBOA is smaller, meaning its competitiveness, universality, and stability for complex optimization algorithms with different characters.

## 4. The Application for Real-World Optimization Problems

In addition, the test functions of CEC2022, the performances of novel algorithms must be verified in solving real-world optimization problems [43]. In this section, five typical engineering optimization problems are used to test the abilities of MISBOA and other algorithms, firstly [44]. Then, the MISBOA is employed to solve a shape optimization problem of combined curves with multiple shape parameters. Based on the 8 comparison algorithms in Section 4, four popular algorithms widely used in practice are added, which are Harris hawks optimization (HHO) [45], walrus optimizer (WO) [46], grey wolf optimization algorithm (GWO) [14], and whale optimization algorithm (WOA) [47].

### 4.1. Engineering Optimization Problems

The results will be recorded after 10 independent runs for each algorithm. For all problems, the size of the population and maximum iteration are 50 and 300, respectively. To fairly measure the performance of different algorithms, the best value, average value, worst value, and standard deviation (Std) results after 10 runs will be calculated. Meanwhile, based on the average value, the ranking result for each algorithm can be determined to illustrate its combination performance.

#### 4.1.1. Step-Cone Pulley Design Problem

**Figure 16:**
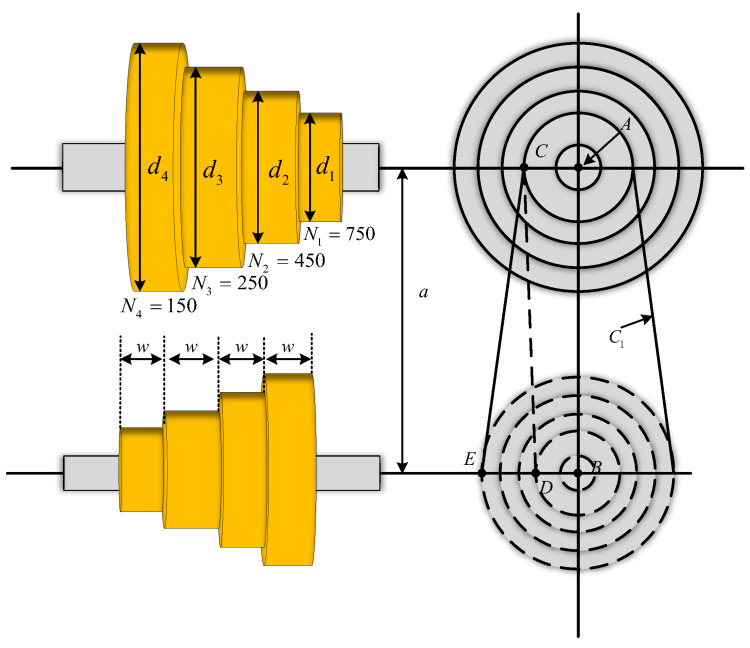
The construction of step-cone pulley design.

The main goal of this problem is to minimize the weight of the four stepped cone pulleys by adjusting five key variables, which are the diameter of each pulley (*d*_1_, *d*_2_, *d*_3_, *d*_4_) and the width of the pulley (*w*). As shown in Figure 16, the system contains 11 nonlinear constraints to ensure that the transmitted power must be at 0.75 hp. Its mathematical model is defined in Equation (24).

$$\vec{x}=[x_{1},x_{2},x_{3},x_{4},x_{5}]=[d_{1},d_{2},d_{3},d_{4},w],$$

(24) $$f(\vec{x})=pw\left\{d_{1}^{2}\left[11+{\left(\frac{N_{1}}{N}\right)}^{2}\right]+d_{2}^{2}\left[1+{\left(\frac{N_{2}}{N}\right)}^{2}\right]+d_{3}^{2}\left[1+{\left(\frac{N_{3}}{N}\right)}^{2}\right]+d_{4}^{2}\left[1+{\left(\frac{N_{4}}{N}\right)}^{2}\right]\right\},$$

$$\begin{array}{l} h_{1}(\vec{x})=C_{1}-C_{2}=0,\\ h_{2}(\vec{x})=C_{1}-C_{3}=0,\\ h_{3}(\vec{x})=C_{1}-C_{4}=0,\\ g_{i}=-R_{i}-2\leq 0,i=1,2,3,4,\\ h_{i}=(0.75\times 745.6998)-P_{i}\leq 0,i=1,2,3,4,\\ C_{i}=\frac{\pi d_{i}}{2}\left(1+\frac{N_{i}}{N}\right)+\frac{{\left(\frac{N_{i}}{N}-1\right)}^{2}}{4a}+2a,i=1,2,3,4,\\ R_{i}=\exp\left\{u\left[\pi -2\sin^{-1}\left(\frac{N_{i}}{N}-1\right)\frac{d_{i}}{2a}\right]\right\},i=1,2,3,4,\\ P_{i}=stw(1-R_{i})\frac{\pi d_{i}N_{i}}{60},i=1,2,3,4,\\ t=8\;\mathrm{mm},s=1.75\;\mathrm{MPa},u=0.35,\rho =7200\;{\mathrm{kg}/m}^{3},a=3\;\mathrm{mm},\\ N_{1}=750,N_{2}=450,N_{3}=250,N_{4}=150,N=350,\\ 0\leq d_{1},d_{2}\leq 60,0\leq d_{3},d_{4},w\leq 60. \end{array}$$

Table 6 summarizes the results of MISBOA and others for the step-cone pulley design problem. Observing the index of best value, the MISBOA and SBOA obtain the same fitness values, which are better than others. However, for the index of worst value, the SBOA is inferior to MISBOA, meaning the stability of MISBOA is enhanced by introducing multiple effective, improved strategies. Thus, for the final ranking results, the proposed MISBOA performs excellently. Smaller Std also supports the conclusion that the MISBOA is not easy to be affected by accidental factors when solving engineering optimization problems. Table 7 lists the best variables obtained by various algorithms for this design problem according to the best fitness value in Table 6.

#### 4.1.2. Planetary Gear Train Design Problem

**Figure 17:**
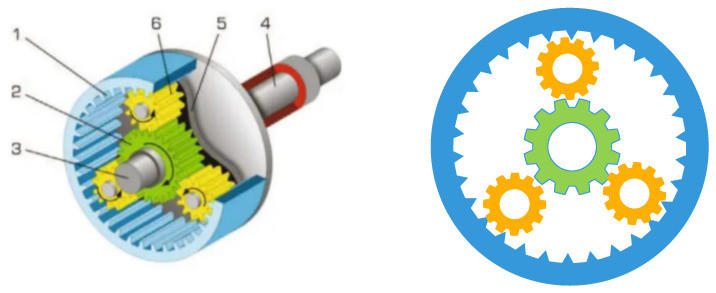
The construction of the planetary gear train design.

The main aim of the planetary gear train design optimization problem is to minimize the maximum error of the transmission ratio used in automobiles. To achieve the target, 9 variables are needed to be adjusted, as shown in Figure 17. In these variables, 6 integer variables (*N*_1_, *N*_2_, *N*_3_, *N*_4_, *N*_5_, *N*_6_) represent the number of the gear teeth, and 3 discrete variables represent the number of gear modulus (*m*_1_, *m*_2_) and the number of planetary gear teeth (*p*). The established model of the planetary gear train design system is shown in Equation (25).

$$\vec{x}=[x_{1},x_{2},x_{3},x_{4},x_{5},x_{6},x_{7},x_{8},x_{9}]=[N_{1},N_{2},N_{3},N_{4},N_{5},N_{6},p,m_{1},m_{2}],$$

(25) $$f(\vec{x})=\max\left|i_{k}-i_{ok}\right|,k=\{1,2,\cdots ,R\},$$

$$\begin{array}{l} i_{1}=\frac{N_{6}}{N_{4}},i_{o1}=3.11,i_{2}=\frac{N_{6}(N_{1}N_{3}+N_{2}N_{4})}{N_{1}N_{3}(N_{6}+N_{4})},i_{oR}=-3.11,i_{R}=-\frac{N_{2}N_{6}}{N_{1}N_{3}},i_{o2}=1.84,\\ g_{1}(\vec{x})=m_{2}(N_{6}+2.5)-D_{\max}\leq 0,\\ g_{2}(\vec{x})=m_{1}(N_{1}+N_{2})+m_{1}(N_{2}+2)-D_{\max}\leq 0,\\ g_{3}(\vec{x})=m_{2}(N_{4}+N_{5})+m_{2}(N_{5}+2)-D_{\max}\leq 0,\\ g_{4}(\vec{x})=\left|m_{1}(N_{1}+N_{2})+m_{2}(N_{6}+N_{3})\right|-m_{1}-m_{2}\leq 0,\\ g_{5}(\vec{x})=-(N_{1}+N_{2})\sin(\pi /p)+N_{2}+2+\delta_{22}\leq 0,\\ g_{6}(\vec{x})=-(N_{6}-N_{3})\sin(\pi /p)+N_{3}+2+\delta_{33}\leq 0,\\ g_{7}(\vec{x})=-(N_{4}+N_{5})\sin(\pi /p)+N_{5}+2+\delta_{55}\leq 0,\\ g_{8}(\vec{x})={\left(N_{3}+N_{5}+2+\delta_{35}\right)}^{2}-{\left(N_{6}-N_{3}\right)}^{2}-{\left(N_{4}+N_{5}\right)}^{2}\\ \;+2(N_{6}-N_{3})(N_{4}+N_{5})\cos\left(\frac{2\pi }{p}-\beta \right)\leq 0,\\ g_{9}(\vec{x})=N_{4}-N_{6}+2N_{5}+2\delta_{56}+4\leq 0,\\ g_{10}(\vec{x})=2N_{3}-N_{6}+N_{4}+2\delta_{34}+4\leq 0,\\ h_{1}(\vec{x})=\frac{N_{6}-N_{4}}{p},\\ \delta_{22}=\delta_{33}=\delta_{55}=\delta_{56}=0.5,\beta =\frac{\cos^{-1}\left({\left(N_{4}+N_{5}\right)}^{2}+{\left(N_{6}-N_{3}\right)}^{2}-{\left(N_{3}+N_{5}\right)}^{2}\right)}{2(N_{6}-N_{3})(N_{4}+N_{5})},\\ p=\{3,4,5\},\;m_{1},m_{2}=\{1.75,\;2.0,\;2.25,\;2.5,\;2.75,\;3.0\},\\ D_{\max}=220,\\ 17\leq N_{1}\leq 96,\;14\leq N_{2}\leq 54,\;14\leq N_{3}\leq 51,\;17\leq N_{4}\leq 46,\;14\leq N_{5}\leq 51,\;48\leq N_{6}\leq 124. \end{array}$$

In Table 8, the solving results of MISBOA and others are shown for the design problem of the planetary gear train design. For the index of best value, the BKA and WOA perform better than the MISBOA, but the MISBOA’s average value and Std have obvious advantages, which lead to the better ranking result. It illustrates the superior comprehensive performance of the MISBOA. It is not reliable if an algorithm can achieve better solutions for only several runs but perform poorly for lots of experiments. Thus, especially for engineering optimization problems, the MISBOA is competitive due to its stronger stability. Table 9 shows the best variables of selected algorithms according to the best solution.

#### 4.1.3. Robot Gripper Design Problem

**Figure 18:**
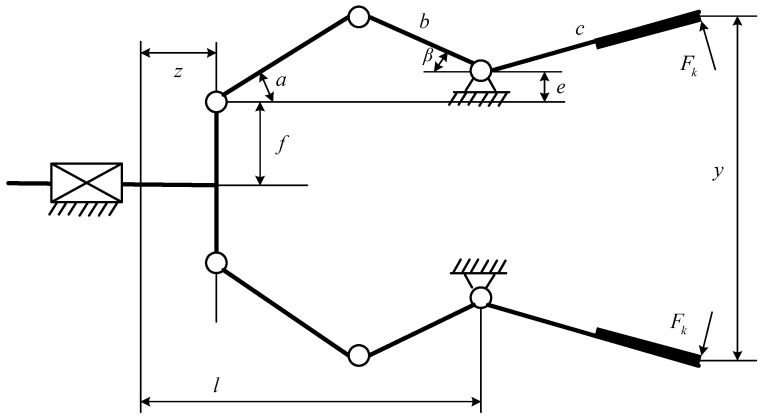
The construction of the robot gripper design.

The problem of robot gripper is a popular optimization problem in mechanical structure engineering. As shown in Figure 18, this system consists of 6 factors, which are connecting rod length, geometric angle between connecting rods, vertical displacement, clamping pressure, actuator displacement of the robotic gripper, and horizontal displacement. 7 key parameters influence the performance of the system, which are three links (*a*, *b*, *c*), the vertical distance between the first robotic arm node and the actuator end *f*, the vertical displacement of the links *e*, the geometric angle between the second and third links *d*, and the horizontal distance between the actuator end and the links node *l*. By adjusting these key parameters, the minimum value of the difference between the minimum and maximum force needs to be obtained. This problem can be described by Equation (26).

$$\vec{x}=[x_{1},x_{2},x_{3},x_{4},x_{5},x_{6},x_{7}]=[a,b,c,e,f,l,d],$$

(26) $$f(\vec{x})=-\underset{z}{\min}F_{k}(x,z)+\underset{z}{\max}F_{k}(x,z),$$

$$\begin{array}{l} g_{1}(\vec{x})=-Y_{\min}+y(\vec{x},Z_{\max})\leq 0,\\ g_{2}(\vec{x})=-y(x,Z_{\max})\leq 0,\\ g_{3}(\vec{x})=Y_{\max}-y(\vec{x},0)\leq 0,\\ g_{4}(\vec{x})=y(\vec{x},0)-Y_{G}\leq 0,\\ g_{5}(\vec{x})=l^{2}+e^{2}-{\left(a+b\right)}^{2}\leq 0,\\ g_{6}(\vec{x})=b^{2}-{\left(a-e\right)}^{2}-{\left(l-Z_{\max}\right)}^{2}\leq 0,\\ g_{7}(\vec{x})=Z_{\max}-l\leq 0,\\ \alpha =\cos^{-1}\left(\frac{a^{2}+g^{2}-b^{2}}{2ag}\right)+\phi ,g=\sqrt{e^{2}+{\left(z-l\right)}^{2}},\\ \beta =\cos^{-1}\left(\frac{b^{2}+g^{2}-a^{2}}{2bg}\right)-\phi ,g=\tan^{-1}\left(\frac{e}{l-z}\right),\\ y(\vec{x},z)=2(f+e+c\sin(\beta +d)),F_{k}(\vec{x},z)=\frac{Pb\sin(\alpha +\beta )}{2c\cos(\alpha )}\\ Y_{\min}=50,Y_{\max}=100,Y_{G}=150,Z_{\max}=100,P=100,\\ 0\leq e\leq 50,\;\;\;\;100\leq c\leq 200,\;\;\;\;10\leq f,a,b\leq 150,\;\;\;\;1\leq d\leq 3.14,100\leq l\leq 300. \end{array}$$

Table 10 provides the results of various algorithms for the robot gripper design problem. For this complex system, the MISBOA achieves the advantages on all four measure indexes. Thus, the MISBOA can be regarded as an effective and reliable approach to solve the design problem of the robot gripper. This kind of preponderance comes from the suitable and reasonable combination of various improved strategies, which leads to better performance in balancing the abilities between exploration and development. Table 11 shows the best control parameters in the robot gripper system according to the best fitness value.

#### 4.1.4. Four-Stage Gearbox Design Problem

**Figure 19:**
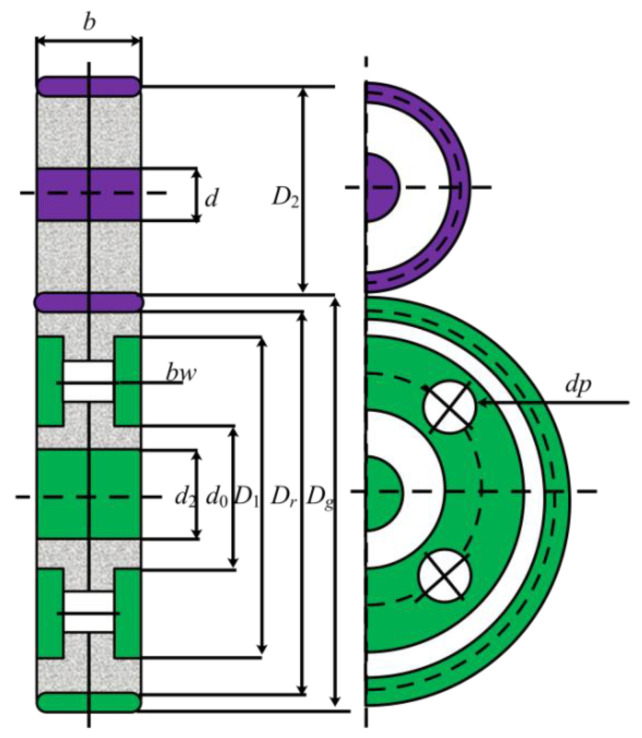
The construction of the four-stage gearbox design problem.

In this problem, researchers must reduce the total weight of the gearbox system by optimizing 22 key variables, as shown in Figure 19. These parameters can be divided into four classes, including the positions of the gears, and pinions, the thickness of the blanks, and the number of teeth. Meanwhile, 86 constraint conditions need to be satisfied, increasing the difficulty of this problem. These requirements involve the contact ratio, pitch, strength of the gears, assembly of gears, kinematics, and size of the gears. The model can be represented by the following:

$$\vec{x}=[x_{1},x_{2},x_{3},\ldots ,x_{22}]=[N_{p1},\ldots ,N_{p4},N_{g1},\ldots ,N_{g4},b_{1}\ldots ,b_{4},x_{p1},x_{g1},\ldots ,x_{g4},y_{p1},y_{g1},\ldots ,y_{g4}],$$

(27) $$f(\vec{x})=\left(\frac{\pi }{1000}\right)\sum_{i=1}^{4}\frac{b_{i}c_{i}^{2}(N_{pi}^{2}+N_{gi}^{2})}{{\left(N_{pi}+N_{gi}\right)}^{2}},$$

$$g_{1}(x)=\left(\frac{366000}{\pi w_{1}}+\frac{2c_{1}N_{p1}}{N_{p1}+N_{g1}}\right)\left(\frac{{\left(N_{p1}+N_{g1}\right)}^{2}}{4b_{1}c_{1}^{2}N_{p1}}\right)-\frac{\sigma_{N}J_{R}}{0.0167WK_{o}K_{m}}\leq 0,$$

$$g_{2}(x)=\left(\frac{366000N_{g1}}{\pi w_{1}N_{p1}}+\frac{2c_{2}N_{p2}}{N_{p2}+N_{g2}}\right)\left(\frac{{\left(N_{p2}+N_{g2}\right)}^{2}}{4b_{2}c_{2}^{2}N_{p2}}\right)-\frac{\sigma_{N}J_{R}}{0.0167WK_{o}K_{m}}\leq 0,$$

$$g_{3}(x)=\left(\frac{366000N_{g1}N_{g2}}{\pi w_{1}N_{p1}N_{p2}}+\frac{2c_{3}N_{p3}}{N_{p3}+N_{g3}}\right)\left(\frac{{\left(N_{p3}+N_{g3}\right)}^{2}}{4b_{3}c_{3}^{2}N_{p3}}\right)-\frac{\sigma_{N}J_{R}}{0.0167WK_{o}K_{m}}\leq 0,$$

$$g_{4}(x)=\left(\frac{366000N_{g1}N_{g2}N_{g3}}{\pi w_{1}N_{p1}N_{p2}N_{p3}}+\frac{2c_{4}N_{p4}}{N_{p4}+N_{g4}}\right)\left(\frac{{\left(N_{p4}+N_{g4}\right)}^{2}}{4b_{4}c_{4}^{2}N_{p4}}\right)-\frac{\sigma_{N}J_{R}}{0.0167WK_{o}K_{m}}\leq 0,$$

$$g_{5}(x)=\left(\frac{366000}{\pi w_{1}}+\frac{2c_{1}N_{p1}}{N_{p1}+N_{g1}}\right)\left(\frac{{\left(N_{p1}+N_{g1}\right)}^{3}}{4b_{1}c_{1}^{2}N_{g1}N_{p1}^{2}}\right)-\left(\frac{\sigma_{H}}{C_{p}}\right)\left(\frac{\sin\left(\varphi \right)\cos\left(\varphi \right)}{0.0334WK_{o}K_{m}}\right)\leq 0,$$

$$g_{6}(x)=\left(\frac{366000N_{g1}}{\pi w_{1}N_{p1}}+\frac{2c_{2}N_{p2}}{N_{p2}+N_{g2}}\right)\left(\frac{{\left(N_{p2}+N_{g2}\right)}^{3}}{4b_{2}c_{2}^{2}N_{g2}N_{p2}^{2}}\right)-\left(\frac{\sigma_{H}}{C_{p}}\right)\left(\frac{\sin\left(\varphi \right)\cos\left(\varphi \right)}{0.0334WK_{o}K_{m}}\right)\leq 0,$$

$$g_{7}(x)=\left(\frac{366000N_{g1}N_{g2}}{\pi w_{1}N_{p1}N_{p2}}+\frac{2c_{3}N_{p3}}{N_{p3}+N_{g3}}\right)\left(\frac{{\left(N_{p3}+N_{g3}\right)}^{3}}{4b_{3}c_{3}^{2}N_{g3}N_{p3}^{2}}\right)-\left(\frac{\sigma_{H}}{C_{p}}\right)\left(\frac{\sin\left(\varphi \right)\cos\left(\varphi \right)}{0.0334WK_{o}K_{m}}\right)\leq 0,$$

$$g_{8}(x)=\left(\frac{366000N_{g1}N_{g2}N_{g3}}{\pi w_{1}N_{p1}N_{p2}N_{p3}}+\frac{2c_{4}N_{p4}}{N_{p4}+N_{g4}}\right)\left(\frac{{\left(N_{p4}+N_{g4}\right)}^{3}}{4b_{4}c_{4}^{2}N_{g4}N_{p4}^{2}}\right)-\left(\frac{\sigma_{H}}{C_{p}}\right)\left(\frac{\sin\left(\varphi \right)\cos\left(\varphi \right)}{0.0334WK_{o}K_{m}}\right)\leq 0,$$

$$\begin{array}{l} g_{9}(x)-g_{12}(x)=-N_{pi}\sqrt{\frac{\sin^{2}(\varphi )}{4}-\frac{1}{N_{pi}}+{\left(\frac{1}{N_{pi}}\right)}^{2}}+N_{pi}\sqrt{\frac{\sin^{2}(\varphi )}{4}-\frac{1}{N_{pi}}{\left(\frac{1}{N_{pi}}\right)}^{2}}\\ +\frac{\sin(\varphi )\left(N_{pi}+N_{gi}\right)}{2}+CR_{\min}\pi \cos(\varphi )\leq 0, \end{array}$$

$$g_{13}(x)-g_{16}(x)=d_{\min}-\frac{2c_{i}N_{pi}}{N_{pi}+N_{gi}}\leq 0,g_{17}(x)-g_{20}(x)=d_{\min}-\frac{2c_{i}N_{gi}}{N_{pi}+N_{gi}}\leq 0,$$

$$g_{21}(x)=x_{p1}+\left(\frac{\left(N_{p1}+2\right)c_{1}}{N_{p1}+N_{g1}}\right)-L_{\max}\leq 0,$$

$$g_{22}(x)-g_{24}(x)=-L_{\max}{\left(\frac{\left(N_{pi}+2\right)c_{i}}{N_{pi}+N_{gi}}\right)}_{i=2,3,4}+x_{g(i-1)}\leq 0,$$

$$g_{25}(x)=-x_{p1}+\left(\frac{\left(N_{p1}+2\right)c_{1}}{N_{p1}+N_{g1}}\right)\leq 0,g_{26–28}(x)=\left(\frac{\left(N_{pi}+2\right)c_{i}}{N_{pi}+N_{gi}}-x_{g(i-1)}\right)\leq 0,$$

$$g_{29}(x)=y_{p1}+\left(\frac{\left(N_{p1}+2\right)c_{1}}{N_{p1}+N_{g1}}\right)-L_{\max}\leq 0,$$

$$g_{30–32}(x)=-L_{\max}+{\left(\frac{\left(N_{pi}+2\right)c_{i}}{N_{pi}+N_{gi}}-y_{g(i-1)}\right)}_{i=2,3,4}\leq 0,$$

$$g_{33}(x)=\frac{\left(N_{p1}+2\right)c_{1}}{N_{p1}+N_{g1}}-y_{p1}\leq 0,g_{34–36}(x)={\left(\frac{\left(N_{pi}+2\right)c_{i}}{N_{pi}+N_{gi}}-y_{g(i-1)}\right)}_{i=2,3,4}\leq 0,$$

$$g_{37–40}(x)=-L_{\max}+\frac{\left(N_{pi}+2\right)c_{i}}{N_{pi}+N_{gi}}+x_{gi}\leq 0,g_{41–44}(x)=-x_{gi}+\left(\frac{\left(N_{pi}+2\right)c_{i}}{N_{pi}+N_{gi}}\right)\leq 0,$$

$$g_{45–48}(x)=y_{gi}+\frac{\left(N_{pi}+2\right)c_{i}}{N_{pi}+N_{gi}}-L_{\max}\leq 0,g_{49–52}(x)=-y_{gi}+\left(\frac{\left(N_{pi}+2\right)c_{i}}{N_{pi}+N_{gi}}\right)\leq 0,$$

$$g_{53–56}(x)=\left(b_{i}-8.255\right)\left(b_{i}-5.715\right)\left(b_{i}-12.70\right)\left(-N_{pi}+0.945c_{i}-N_{gi}\right)\left(-1\right)\leq 0,$$

$$g_{57–60}(x)=\left(b_{i}-8.255\right)\left(b_{i}-3.175\right)\left(b_{i}-12.70\right)\left(-N_{pi}+0.646c_{i}-N_{gi}\right)\leq 0,$$

$$g_{61–64}(x)=\left(b_{i}-5.715\right)\left(b_{i}-3.175\right)\left(b_{i}-12.70\right)\left(-N_{pi}+0.504c_{i}-N_{gi}\right)\leq 0,$$

$$g_{65–68}(x)=\left(b_{i}-5.715\right)\left(b_{i}-3.175\right)\left(b_{i}-8.255\right)\left(-N_{pi}-N_{gi}\right)\leq 0,$$

$$g_{69–72}(x)=\left(b_{i}-8.255\right)\left(b_{i}-5.715\right)\left(b_{i}-12.70\right)\left(N_{pi}+N_{gi}-1.812c_{i}\right)\left(-1\right)\leq 0,$$

$$g_{73–76}(x)=\left(b_{i}-8.255\right)\left(b_{i}-3.175\right)\left(b_{i}-12.70\right)\left(N_{pi}+N_{gi}-0.945c_{i}\right)\leq 0,$$

$$g_{77–80}(x)=\left(b_{i}-5.715\right)\left(b_{i}-3.175\right)\left(b_{i}-12.70\right)\left(N_{pi}+N_{gi}-0.646c_{i}\right)\left(-1\right)\leq 0,$$

$$g_{81–84}(x)=\left(b_{i}-5.715\right)\left(b_{i}-3.175\right)\left(b_{i}-8.255\right)\left(N_{pi}+N_{gi}-0.504c_{i}\right)\leq 0,$$

$$g_{85}(x)=w_{\min}-\frac{w_{1}\left(N_{p1}N_{p2}N_{p3}N_{p4}\right)}{N_{g1}N_{g2}N_{g3}N_{g4}}\leq 0,g_{86}(x)=-w_{\max}\frac{w_{1}\left(N_{p1}N_{p2}N_{p3}N_{p4}\right)}{N_{g1}N_{g2}N_{g3}N_{g4}}\leq 0,$$

$$c_{i}=\sqrt{{\left(y_{gi}-y_{p1}\right)}^{2}+{\left(x_{gi}-x_{p1}\right)}^{2}},K_{o}=1.5,d_{\min}=25,J_{R}=0.2,$$

$$\varphi =120,W=55.9,K_{m}=1.6,CR_{\min}=1.4,L_{\max}=127,C_{p}=464,$$

$$\sigma_{H}=3290,w_{\max}=255,w_{1}=5000,\sigma_{N}=2090,w_{\min}=245,$$

$$b_{i}\in \left\{3.175,12.7,8.255,5.715\right\},7\leq N_{gi},N_{pi}\leq 76,$$

$$y_{pi},x_{pi},y_{gi},x_{gi}\in \left\{12.7,38.1,25.4,50.8,76.2,63.5,88.9,114.3,101.6\right\}.$$

Table 12 includes the solving results of all algorithms for the four-stage gearbox design problem. From the results, it can be noticed that the performance of different algorithms varies a lot due to the increase of variables and the introduction of lots of constraints. For example, though the SBOA achieves the best value after 10 runs, its average value is poor compared with the MISBOA. Compared with other algorithms, the advantage of MISBOA on the index of average value is further expanded. Thus, the MISBOA is a suitable tool for this complex problem. Table 13 shows the best variables according to the best fitness value of each algorithm.

#### 4.1.5. Traveling Salesman Problem

The traveling salesman problem (TSP) is a well-known combinatorial optimization problem. In this problem, each salesman needs to find a path in a certain number of cities to make the total length of the shortest path. Meanwhile, the planned path has to satisfy the requirement, which passes through each city once and only once and finally returns to the starting point. TSP is widely applied for practical applications, such as logistics, transportation, and other fields. Because the candidate solutions to this problem are the full permutation of all cities, the difficulty of this problem will increase dramatically with the increase in the number of cities.

**Figure 20:**
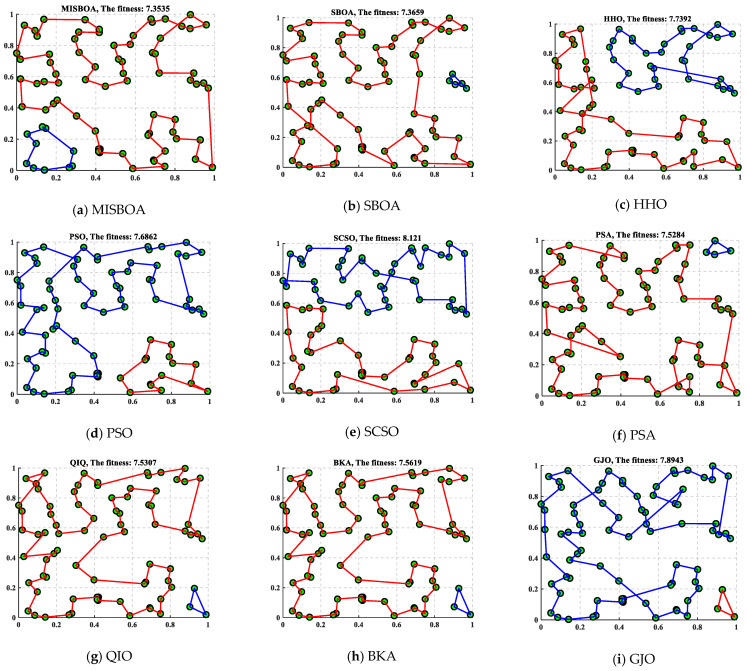

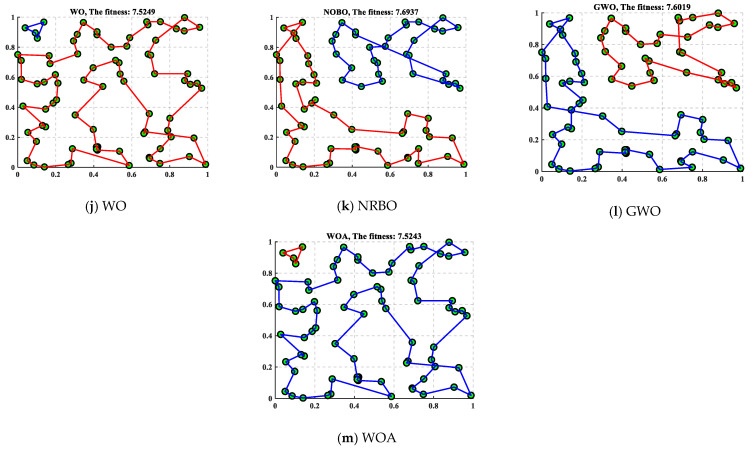
The best results for TSP with 80 cities and 2 traveling salesman.

In this case, there are 80 randomly generated cities and 2 traveling salesmen. Table 14 includes the results obtained by MISBOA and others for the TSP with 80 cities and 2 traveling salesmen. For the indexes of the best, average, and standard, the MISBOA ranks first. That is, for complex problems with lots of variables, the MISBOA can reasonably search for the feasible space with the suitable strategies in different stages. For the original SBOA, the lack of more effective searching strategies leads to its worse overall performance compared with the MISBOA. Figure 20 plots the best routes for travel salesman obtained by different algorithms and their convergence curves and the box plots. Comparing Figure 20a,b, the number of cities visited by the two salesmen is not evenly balanced. Compared with other solutions, the route of MISBOA is more reasonable, which avoids additional consumption. However, routes based on other algorithms are wound together, leading to longer total distance.

### 4.2. Shape Optimization Problems of Combined Curves

In the field of practical engineering applications, it is of great significance to generate a smooth curve that can pass through all given data points by the interpolation approach [48]. In this section, the energy of the combined quartic generalized Ball interpolation (CQGBI) curve is studied and optimized.

The CQGBI curve is constructed by combing several quartic curves. For the given data points $Q_{i}\in R^{2}(i=0,\;1,\;\cdots ,\;n)$, a CQGBI curve R(t;λ,μ) can be obtained, as shown in Equation (28) [49].

(28) $$R(t;\lambda ,\mu )=\left\{\begin{array}{l} R_{1}(t;\lambda_{1},\mu_{1}),\\ \vdots \\ R_{1}(t;\lambda_{j},\mu_{j}),\\ \vdots \\ R_{1}(t;\lambda_{n},\mu_{n}), \end{array}\right.$$

where

(29) $$\begin{array}{ll} R_{j}(t;\lambda_{j},\mu_{j}) & =(1+2t){\left(1-t\right)}^{2}P_{j-1}+(3-2t)t^{2}P_{j}\\ & +\frac{(2+\lambda_{j}-\lambda_{j}t)t{\left(1-t\right)}^{2}}{2+\lambda_{j}}P_{j-1}^{\prime }\\ & -\frac{\left(2+\mu_{j}t)t^{2}(1-t\right)}{2+\mu_{j}}P_{j}^{\prime }, \end{array}$$

in which $\lambda_{j},\;\mu_{j}\in (-2,\;4]\;(j=1,2,\ldots ,n)$ are the shape parameters and can be adjusted.

Hence, it is necessary to select more suitable parameters to maintain the smoothness of the obtained combined curve. Here, the curvature variation energy is used to measure the smoothness of the CQGBI curve, which is [50].

(30) $$E_{j}(\lambda_{j},\mu_{j})=\int_{0}^{1}\left\|R_{j}^{\prime \prime \prime }(t;\lambda_{j},\mu_{j})\right\|dt,$$

where $R_{j}(t;\lambda_{j},\mu_{j})$ is the curve of the *j*-th section of the CQGBI curve.

After further calculation, Equation (30) can be represented by

(31) $$R_{j}^{\prime \prime \prime }(t;\lambda_{j},\mu_{j})=12P_{j-1}-12P_{j}+\frac{18\lambda_{j}-24\lambda_{j}t+12}{2+\lambda_{j}}P_{j-1}^{\prime }-\frac{6\mu_{j}-24\mu_{j}t+12}{2+\mu_{j}}P_{j}^{\prime },$$

Combined Equation (30) and Equation (31), we can obtain

(32) $$\begin{array}{ll} E_{j}(\lambda_{j},\mu_{j}) & =\int_{0}^{1}\left\|12P_{j-1}-12P_{j}+\frac{18\lambda_{j}-24\lambda_{j}t+12}{2+\lambda_{j}}P_{j-1}^{\prime }-\frac{6\mu_{j}-24\mu_{j}t+12}{2+\mu_{j}}P_{j}^{\prime }\right\|dt\\ & =144{\left\|P_{j-1}\right\|}^{2}+144{\left\|P_{j}\right\|}^{2}-288P_{j-1}\cdot P_{j}+k_{1}{\left\|P_{j-1}^{\prime }\right\|}^{2}+k_{2}{\left\|P_{j}^{\prime }\right\|}^{2}+k_{3}P_{j-1}\cdot P_{j-1}^{\prime }\\ & +k_{4}P_{j-1}\cdot P_{j}^{\prime }-k_{3}P_{j}\cdot P_{j-1}^{\prime }-k_{4}P_{j}\cdot P_{j}^{\prime }+k_{5}P_{j-1}^{\prime }\cdot P_{j}^{\prime }, \end{array}$$

where $k_{1}=\frac{84\lambda_{j}^{2}-144\lambda_{j}+144}{{\left(2+\lambda_{j}\right)}^{2}}$, $k_{2}=\frac{84\mu_{j}^{2}-144\mu_{j}+144}{{\left(2+\mu_{j}\right)}^{2}}$, $k_{3}=\frac{144\lambda_{j}+288}{2+\lambda_{j}}$, $k_{4}=\frac{144\mu_{j}+288}{2+\mu_{j}}$, $k_{5}=\frac{144\lambda_{j}+144\mu_{j}-24\lambda_{j}\mu_{j}+288}{\left(2+\lambda_{j})(2+\mu_{j}\right)}$.

Then, by regarding the total energy of the whole CQGBI curve, the optimization model can be established by Equation (33).

$$\vec{x}=[x_{1},x_{2},\cdots ,x_{2n}]=[\lambda_{1},\lambda_{2},\cdots ,\lambda_{n},\mu_{1},\mu_{2},\cdots ,\mu_{n}],$$

(33) $$\begin{array}{ll} f(\vec{x}) & =\sum_{j=1}^{n}E_{j}(\lambda_{j},\mu_{j})=\sum_{j=1}^{n}(144{\left\|P_{j-1}\right\|}^{2}+144{\left\|P_{j}\right\|}^{2}-288P_{j-1}\cdot P_{j}+k_{1}{\left\|P_{j-1}^{\prime }\right\|}^{2}+k_{2}{\left\|P_{j}^{\prime }\right\|}^{2}\\ & +k_{3}P_{j-1}\cdot P_{j-1}^{\prime }+k_{4}P_{j-1}\cdot P_{j}^{\prime }-k_{3}P_{j}\cdot P_{j-1}^{\prime }-k_{4}P_{j}\cdot P_{j}^{\prime }+k_{5}P_{j-1}^{\prime }\cdot P_{j}^{\prime }). \end{array}$$

$$\lambda_{j},\;\mu_{j}\in (-2,\;4]\;(j=1,2,\ldots ,n)$$

**Figure 21:**
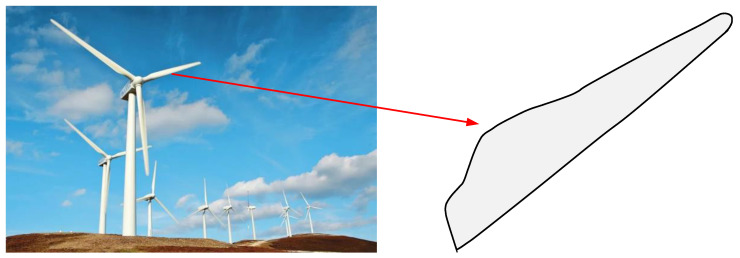
The shape of wind-driven generator blades.

That is, for a CQGBI curve with *n* + 1 data points, there are 2*n* parameters that need to be optimized. Here, the shape optimization problem of wind-driven generator blades is studied. As shown in Figure 21, as a core component of wind power generation systems, the shape of wind-driven generator blades will significantly affect the efficiency of power generation. Thus, the MISBOA and other algorithms are used to select the most suitable parameters to reduce the energy of the CQGBI curve, which describes the shape of a wind-driven generator blade.

**Figure 22:**
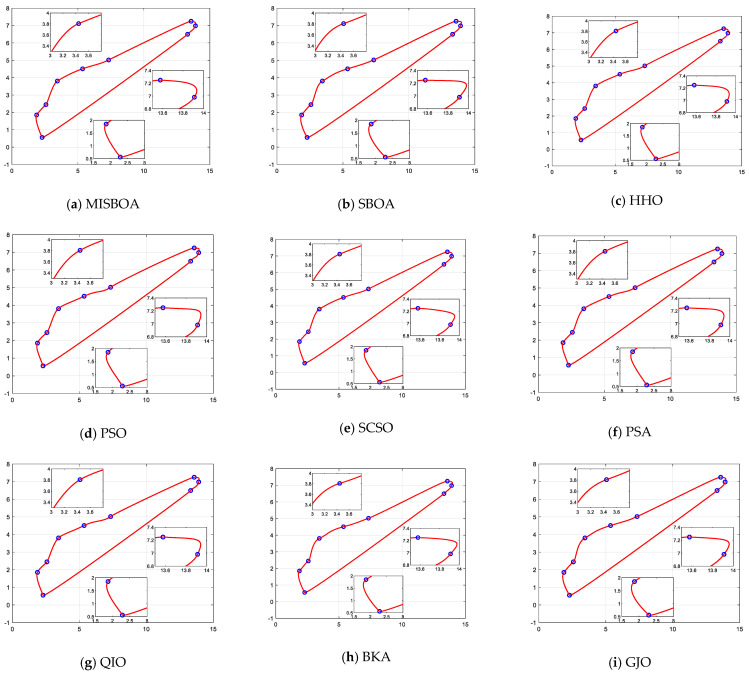

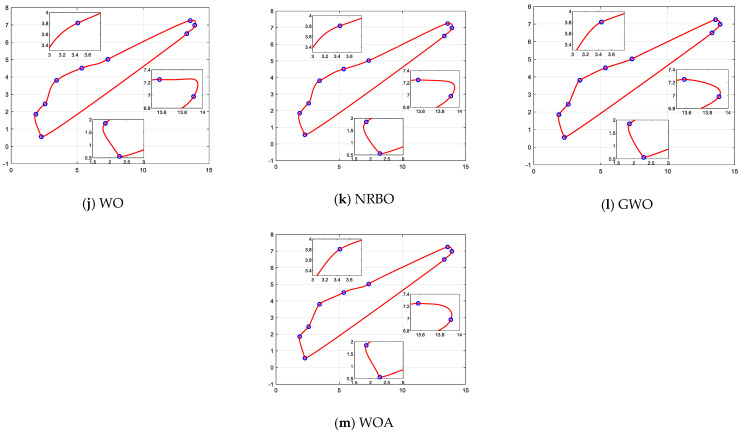
The best design for wind-driven generator blades according to the best solutions of various algorithms.

After 10 times running, the results of various algorithms for the shape optimization problem of wind-driven generator blades are listed in Table 15. From the summarized data, the MISBOA finally ranks first due to its excellent performance on the four measure indexes. Especially for the standard deviation, the MISBOA is significantly superior to other algorithms. That is, it even can achieve similar solutions with higher accuracy for each running, meaning the ability to get better results in fewer execution times. For the original SBOA, its performance is severely affected, only ranking fifth. The QIO and HHO obtain the same best value but have poor comprehensive performance due to the larger average value. Based on the best solution, the best designs for wind-driven generator blades are plotted in Figure 22. Local enlargements of some key sites are also drawn. For the solutions with larger fitness values, curves at corners are not smooth and will affect power generation efficiency, such as Figure 22d,i,l. For the MISBOA, the CQGBI curve is smoother to reduce unnecessary wastage.

## 5. Conclusions and Future Work

Aiming at the defects of the original SBOA algorithm, this paper proposed an improved version by introducing four specific strategies. Firstly, a feedback regulation mechanism based on incremental PID control is applied. It aims to keep higher searching effectiveness by updating the whole population according to the output value. In the hunting stage, to enhance the success rate of capture, a golden sinusoidal guidance strategy is employed. Meanwhile, to keep the population, diversity, a cooperative camouflage strategy, and an update strategy based on cosine similarity are introduced into the escaping stage.

According to the obtained results in solving test functions of the CEC2020 suite. When the dimension is 10, the MISBOA can rank firstly on 6 functions, while the SBOA can rank firstly only on 1 and 4 functions. Meanwhile, the comprehensive performance of each algorithm on different functions is compared by radar map. Though the final rank of QIO is the second, it ranks fourth on 4 functions and only ranks eighth on CEC12, meaning poorer stability than MISBOA. The worst rank of MISBOA is third on only one function. When the dimension is increased to 20, the advantage of MISBOA is more obvious. It ranks first on the rest 10 test functions, accounting for 83.33% of the total. The original SBOA and QIO algorithms rank first on only 1 test function. This difference illustrates the introduction of improvement strategies that effectively enhance the searching accuracy and stability of MISBOA for various problems. From the convergence curves, due to the effect of the feedback regulation mechanism and golden sinusoidal guidance strategy, the MISBOA can quickly approach better solutions. In the later searching process, it also can keep staying ahead because two strategies can help MISBOA escape local optimums. For five real-world optimization problems, the MISBOA also has the best performance on the fitness values, indicating stronger searching ability with higher accuracy and stability. Finally, when it is used to solve the shape optimization problem of the CQGBI curve, the shape can be designed to be smoother according to the obtained parameters based on MISBOA to improve power generation efficiency.

In the future, the practical application of the MISBOA algorithm will be further explored. Meanwhile, whether the improved strategy adopted in this paper will have a positive impact on other original algorithms is also a problem that needs to be studied.

## Figures and Tables

**Table 1:** Parameters of the selected algorithms.

Algorithms	Proposed Time	Parameters
BKA	2024	Parameter *P* = 0.9.
GJO	2022	The constant *c*_1_ = 1.5.
PSO	1995	Cognitive factor *c*_1_ = 2, social factor *c*_2_ = 2.5, acceleration weight *w* = 2.
PSA	2024	Proportion factor *K_p_* = 1, integral factor *K_i_* = 0.5, differential factor *K_d_* = 1.2, levy flight factor of *β* = 1.5.
QIO	2023	None
NRBO	2024	Deciding factor for trap avoidance operator *DF* = 0.6;
SCSO	2022	The maximum sensitivity *S* = 2.
SBOA	2024	Levy flight factor *β* = 1.5.
MISBOA	—	Levy flight factor *β* = 1.5, proportion factor *K_p_* = 1, integral factor *K_i_* = 0.5, differential factor *K_d_* = 1.2.

**Table 2:** Results of different algorithms on CEC2022 functions with 10 dimensions.

	Index	BKA	GJO	PSO	PSA	QIO	NRBO	SCSO	SBOA	MISBOA
CEC01	Ave	320.52	1437.41	322.74	300.00	300.00	424.69	629.84	300.00	300.00
	Std	9.79E+01	1.26E+03	2.05E+01	4.09E-14	4.78E-08	1.31E+02	7.70E+02	4.48E-14	2.59E-14
	Rank	5	9	6	2	4	7	8	3	1
	Time	0.99	0.79	0.36	0.45	3.09	2.75	4.16	1.03	2.75
CEC02	Ave	411.45	438.50	405.57	410.41	406.65	424.36	431.70	404.92	402.98
	Std	20.67	24.18	5.16	20.73	17.63	25.31	31.50	3.54	3.49
	Rank	6	9	3	5	4	7	8	2	1
	Time	1.21	0.93	0.40	0.58	3.54	2.64	4.84	1.03	3.72
CEC03	Ave	615.80	603.44	600.01	600.00	600.00	617.38	611.65	600.00	600.00
	Std	8.88E+00	2.55E+00	8.79E-03	1.84E-05	4.61E-05	6.18E+00	7.01E+00	3.45E-06	0.00E+00
	Rank	8	6	5	3	4	9	7	2	1
	Time	1.50	1.04	0.60	0.77	3.37	2.28	4.01	1.33	3.42
CEC04	Ave	813.63	825.17	814.03	822.84	810.61	820.18	826.31	809.59	807.33
	Std	4.95	7.31	5.95	13.24	4.00	7.80	5.86	4.55	3.26
	Rank	4	8	5	7	3	6	9	2	1
	Time	1.50	1.04	0.60	0.77	3.37	2.28	4.01	1.33	3.42
CEC05	Ave	995.50	936.73	900.19	915.27	900.18	968.53	1009.84	900.00	900.00
	Std	6.85E+01	3.62E+01	2.33E-01	2.80E+01	4.52E-01	7.16E+01	1.35E+02	2.70E-13	0.00E+00
	Rank	8	6	4	5	3	7	9	2	1
	Time	1.06	0.85	0.41	0.59	3.50	3.64	4.74	1.05	2.71
CEC06	Ave	2086.69	7083.45	3478.84	3362.98	1801.11	1991.39	4725.88	3282.25	1825.88
	Std	1118.41	1627.54	2632.62	1543.53	0.76	328.28	1984.53	1436.34	16.18
	Rank	4	9	7	6	1	3	8	5	2
	Time	1.16	1.02	0.46	0.65	3.51	5.10	5.95	1.01	3.09
CEC07	Ave	2035.31	2037.73	2013.24	2018.92	2002.84	2044.48	2037.13	2006.41	2003.56
	Std	10.63	14.08	9.75	5.38	3.79	15.56	12.63	8.91	6.71
	Rank	6	8	4	5	1	9	7	3	2
	Time	2.35	1.66	1.07	1.18	4.91	10.88	7.22	2.02	5.90
CEC08	Ave	2221.96	2225.92	2214.48	2222.14	2211.81	2229.52	2226.38	2211.36	2206.67
	Std	8.03	4.32	9.79	20.97	10.54	4.98	4.35	10.26	9.24
	Rank	5	7	4	6	3	9	8	2	1
	Time	1.86	1.20	0.80	1.01	3.38	3.51	4.18	1.84	4.63
CEC09	Ave	2543.42	2558.71	2533.31	2529.28	2529.28	2531.31	2549.56	2529.28	2529.28
	Std	43.08	22.24	1.06	0.00	0.00	3.15	23.20	0.00	0.00
	Rank	7	9	6	1	4	5	8	2	3
	Time	1.57	1.05	0.63	0.81	3.41	3.25	4.18	1.47	3.55
CEC10	Ave	2543.71	2553.59	2542.78	2528.25	2500.35	2556.39	2533.86	2525.53	2507.38
	Std	57.93	62.08	56.88	51.23	0.07	64.70	56.12	46.63	27.46
	Rank	7	8	6	4	1	9	5	3	2
	Time	1.48	1.00	0.58	0.76	3.25	3.39	4.09	1.34	3.68
CEC11	Ave	2659.40	2822.80	2713.43	2727.86	2600.00	2816.16	2731.96	2638.35	2623.33
	Std	124.56	179.32	160.75	173.53	0.00	159.55	158.19	104.80	89.76
	Rank	4	9	5	6	1	8	7	3	2
	Time	2.27	1.47	1.04	1.17	4.47	3.97	5.15	2.20	5.52
CEC12	Ave	2864.74	2865.07	2868.81	2865.27	2866.15	2865.21	2865.38	2860.36	2862.03
	Std	1.80	2.36	1.12	1.96	1.83	1.28	1.92	1.60	1.67
	Rank	3	4	9	6	8	5	7	1	2
	Time	3.16	1.99	1.34	1.69	6.36	12.41	7.26	3.43	7.26
Average rank		5.58	7.67	5.33	4.67	3.08	7.00	7.58	2.50	1.58
Finial rank		6	9	5	4	3	7	8	2	1
Average time		1.68	1.17	0.69	0.87	3.85	4.68	4.98	1.59	4.14

**Table 3:** The *p*-value of WRST between the MISBOA and other algorithms on CEC2022 with 10 dimensions.

	BKA	GJO	PSO	PSA	QIO	NRBO	SCSO	SBOA
CEC01	6.32E-12/+	6.32E-12/+	6.32E-12/+	8.42E-09/+	6.32E-12/+	6.32E-12/+	6.32E-12/+	1.43E-02/+
CEC02	2.82E-04/+	3.10E-10/+	1.67E-03/+	1.64E-03/+	5.18E-02/=	1.41E-08/+	1.37E-06/+	3.66E-04/+
CEC03	3.15E-12/+	3.15E-12/+	3.15E-12/+	9.18E-08/+	3.15E-12/+	3.15E-12/+	3.15E-12/+	1.58E-02/+
CEC04	6.88E-07/+	4.78E-11/+	4.04E-06/+	2.28E-08/+	4.17E-04/+	1.36E-09/+	3.21E-11/+	4.24E-02/+
CEC05	1.21E-12/+	1.21E-12/+	1.21E-12/+	1.21E-12/+	1.21E-12/+	1.21E-12/+	1.21E-12/+	9.04E-13/+
CEC06	3.82E-09/+	3.02E-11/+	7.39E-11/+	5.07E-10/+	3.02E-11/−	1.78E-10/+	3.02E-11/+	3.34E-11/+
CEC07	5.49E-11/+	3.02E-11/+	1.58E-04/+	2.19E-08/+	5.87E-04/−	4.50E-11/+	4.50E-11/+	2.71E-02/+
CEC08	2.67E-09/+	7.39E-11/+	1.43E-05/+	5.09E-06/+	5.83E-03/+	3.02E-11/+	7.39E-11/+	5.94E-02/+
CEC09	5.73E-11/+	1.21E-12/+	1.21E-12/+	2.78E-05/−	1.21E-12/+	1.21E-12/+	1.21E-12/+	3.34E-01/=
CEC10	3.50E-09/+	1.07E-09/+	2.67E-09/+	2.67E-09/+	1.43E-08/−	8.10E-10/+	2.03E-09/+	2.00E-06/=
CEC11	1.16E-09/+	4.85E-10/+	5.35E-10/+	1.29E-08/+	1.88E-09/−	3.98E-10/+	8.71E-10/+	8.32E-03/+
CEC12	4.62E-08/+	4.62E-08/+	2.96E-11/+	1.14E-07/+	2.11E-10/+	1.26E-09/+	4.92E-09/+	2.25E-03/−
+/=/−	12/0/0	12/0//0	12/0/0	11/0/1	7/1/4	12/0/0	12/0/0	9/2/1

**Table 4:** Results of different algorithms on CEC2022 functions with 20 dimensions.

	Index	BKA	GJO	PSO	PSA	QIO	NRBO	SCSO	SBOA	MISBOA
CEC01	Ave	791.30	11,801.49	4525.72	300.00	473.69	6355.33	7879.45	333.17	300.00
	Std	1562.25	3673.44	1114.88	0.00	178.77	1754.62	3915.38	33.94	0.00
	Rank	5	9	6	2	4	7	8	3	1
	Time	1.01	1.00	0.43	0.69	4.89	4.55	6.98	1.05	3.29
CEC02	Ave	462.82	557.79	480.78	449.38	451.37	588.88	518.37	452.92	440.93
	Std	11.70	88.91	12.19	19.88	19.27	60.80	40.44	8.68	18.84
	Rank	5	8	6	2.00	3	9	7	4	1
	Time	1.09	0.99	0.42	0.68	4.99	3.87	7.14	1.12	2.93
CEC03	Ave	641.17	620.69	602.05	602.12	600.05	647.93	641.33	600.01	600.00
	Std	10.43	10.60	0.73	2.74	0.15	9.47	11.23	0.04	0.00
	Rank	7	6	4	5	3	9	8	2	1
	Time	2.59	1.91	1.25	1.54	6.88	6.27	10.11	2.53	6.26
CEC04	Ave	859.76	887.60	902.92	855.63	836.68	900.91	884.70	827.14	831.58
	Std	12.65	26.30	11.33	17.21	9.00	19.41	14.25	8.67	12.25
	Rank	5	7	9	4	3	8	6	1	2
	Time	1.69	1.33	0.74	1.07	6.43	6.94	21.32	1.79	4.55
CEC05	Ave	1885.84	1623.36	1006.72	1513.93	913.97	2078.83	2109.87	900.03	900.02
	Std	392.09	321.74	98.38	393.05	11.00	437.03	359.83	0.06	0.08
	Rank	7	6	4	5	3	8	9	2	1
	Time	1.62	1.36	0.73	1.10	6.09	8.25	23.38	1.41	3.90
CEC06	Ave	4933.37	4,852,799.47	75,196.95	4601.02	4301.75	10,967.32	125,429.45	7540.34	2350.78
	Std	4780.87	11,904,051.13	200,057.18	3400.84	2503.06	18,029.84	386,929.93	7325.22	881.48
	Rank	4	9	7	3	2	6	8	5	1
	Time	2.44	2.06	0.99	1.44	11.43	8.47	27.42	2.48	6.87
CEC07	Ave	2094.99	2096.21	2045.75	2074.80	2034.29	2130.26	2118.64	2031.69	2026.00
	Std	20.09	37.95	11.40	51.21	13.09	39.39	28.80	9.82	5.86
	Rank	6	7	4	5	3	9	8	2	1
	Time	3.08	1.94	1.35	1.68	6.81	12.40	26.72	2.65	6.89
CEC08	Ave	2249.56	2247.94	2246.59	2254.94	2222.84	2292.85	2247.05	2222.79	2221.58
	Std	45.64	41.35	36.40	52.01	4.84	58.32	34.71	1.65	0.92
	Rank	7	6	4	8	3	9	5	2	1
	Time	3.39	2.15	1.63	1.99	7.02	13.45	27.18	3.04	7.67
CEC09	Ave	2506.20	2551.08	2488.33	2480.78	2480.78	2552.55	2527.15	2480.78	2480.78
	Std	63.53	39.97	1.86	0.00	0.00	39.51	37.19	0.00	0.00
	Rank	6	8	5	3	4	9	7	2	1
	Time	2.84	1.94	1.36	1.78	6.86	11.80	26.20	2.59	6.31
CEC10	Ave	3521.79	3610.37	2944.78	2669.55	2507.00	3380.06	3176.59	2530.12	2522.89
	Std	1013.07	1469.48	465.33	183.17	34.71	1308.25	1051.78	54.78	51.38
	Rank	8	9	5	4	1	7	6	3	2
	Time	2.42	1.68	1.10	1.45	6.45	10.67	22.19	2.11	5.53
CEC11	Ave	3380.09	4052.40	2982.61	2916.67	2935.35	3940.42	3483.37	2952.02	2906.67
	Std	724.80	390.72	126.19	74.66	110.57	290.98	386.07	111.55	94.44
	Rank	6	9	5	2	3	8	7	4	1
	Time	3.48	2.35	1.78	2.22	7.20	13.48	23.89	7.53	3.18
CEC12	Ave	3017.43	2993.92	3016.04	2969.80	2978.36	3009.53	3019.32	2939.76	2900.00
	Std	58.80	37.88	18.18	27.28	20.94	47.79	61.23	4.98	0.00
	Rank	8	5	7	3	4	6	9	2	1
	Time	3.95	2.61	1.97	2.46	8.31	14.44	23.61	3.51	8.39
Average rank		6.17	7.42	5.50	3.83	3.00	7.92	7.33	2.67	1.17
Finial rank		6	8	5	4	3	9	7	2	1
Average time		2.47	1.78	1.15	1.51	6.95	9.55	20.51	2.65	5.48

**Table 5:** The *p*-value of WRST between the MISBOA and other algorithms on CEC2022 with 20 dimensions.

	BKA	GJO	PSO	PSA	QIO	NRBO	SCSO	SBOA
CEC01	3.02E-11/+	3.02E-11/+	3.02E-11/+	1.60E-07/+	3.02E-11/+	3.02E-11/+	3.02E-11/+	3.02E-11/+
CEC02	1.84E-07/+	3.24E-11/+	3.96E-11/+	8.96E-04/+	1.51E-05/+	2.93E-11/+	3.96E-11/+	1.53E-08/+
CEC03	2.27E-11/+	2.27E-11/+	2.27E-11/+	2.27E-11/+	2.27E-11/+	2.27E-11/+	2.27E-11/+	7.47E-09/+
CEC04	7.12E-09/+	1.21E-10/+	3.02E-11/+	2.67E-07/+	3.39E-02/+	3.02E-11/+	3.69E-11/+	1.76E-01/=
CEC05	2.66E-11/+	2.66E-11/+	2.66E-11/+	2.66E-11/+	3.60E-11/+	2.66E-11/+	2.66E-11/+	2.19E-08/+
CEC06	4.42E-06/+	3.69E-11/+	4.57E-09/+	6.38E-03/+	5.87E-04/+	7.74E-06/+	1.20E-08/+	1.49E-06/+
CEC07	3.02E-11/+	3.34E-11/+	1.07E-09/+	2.15E-10/+	2.84E-04/+	3.02E-11/+	3.02E-11/+	1.33E-02/+
CEC08	3.34E-11/+	3.02E-11/+	3.34E-11/+	8.88E-06/+	2.28E-01/=	3.02E-11/+	3.02E-11/+	5.26E-04/+
CEC09	8.87E-12/+	8.87E-12/+	8.87E-12/+	8.87E-12/+	8.87E-12/+	8.87E-12/+	8.87E-12/+	8.86E-12/+
CEC10	8.48E-09/+	1.31E-08/+	1.01E-08/+	3.08E-08/+	6.74E-06/−	7.09E-08/+	2.38E-07/+	8.56E-04/+
CEC11	2.36E-04/+	2.02E-11/+	9.63E-02/=	1.46E-01/=	5.92E-02/=	2.02E-11/+	2.67E-10/+	2.13E-01/=
CEC12	3.02E-11/+	3.02E-11/+	3.02E-11/+	3.02E-11/+	3.02E-11/+	3.02E-11/+	3.02E-11/+	3.02E-11/+
+/=/−	12/0/0	12/0/0	11/1/0	11/1/0	9/2/1	12/0/0	12/0/0	10/2/0

**Table 6:** Results of various algorithms for the step-cone pulley design problem.

Algorithms	The Best	The Average	The Worst	The Std	Rank
MISBOA	9.80015	9.80015	9.80015	8.64E-11	1
SBOA	9.80015	9.80016	9.80019	1.48E-05	2
HHO	9.82369	9.98223	10.30760	1.50E-01	8
PSO	10.05302	10.32013	10.59591	1.82E-01	10
SCSO	10.38607	12.60736	15.24964	1.69E+00	13
PSA	9.80095	9.90024	10.02584	9.08E-02	6
QIO	9.80028	9.80087	9.80183	5.40E-04	3
BKA	9.80709	10.15584	11.20607	5.61E-01	9
GJO	9.85004	9.90851	9.99557	4.78E-02	7
WO	9.80062	9.80902	9.83530	1.09E-02	4
NRBO	9.93519	10.35455	10.83489	2.89E-01	11
GWO	9.81930	9.83887	9.87625	2.37E-02	5
WOA	10.43362	11.85415	14.78914	1.50E+00	12

**Table 7:** The best variables of various algorithms for the step-cone pulley design problem.

Algorithms	*x*_1_ (*d*_1_)	*x*_2_ (*d*_2_)	*x*_3_ (*d*_3_)	*x*_4_ (*d*_4_)	*x*_5_ (*w*)
MISBOA	20.5427	28.2575	50.7967	84.4957	90.0000
SBOA	20.5427	28.2575	50.7967	84.4957	90.0000
HHO	20.5671	28.4052	50.8662	84.5135	89.9810
PSO	20.0943	29.7522	51.2044	85.2100	89.3350
SCSO	18.6324	30.8902	52.2056	86.8985	87.5718
PSA	20.5424	28.2597	50.8006	84.5021	89.9932
QIO	20.5428	28.2577	50.7974	84.4962	89.9998
BKA	20.5745	28.2576	50.8071	84.5254	90.0000
GJO	20.7429	28.3120	50.9868	84.6088	90.0000
WO	20.5445	28.2606	50.7974	84.4975	89.9988
NRBO	22.3892	28.2576	50.7968	84.5012	89.9998
GWO	20.5880	28.3594	50.8323	84.5390	90.0000
WOA	24.8581	29.2136	53.0311	85.2885	89.1611

**Table 8:** Results of various algorithms for the planetary gear train design problem.

Algorithms	The Best	The Average	The Worst	The Std	Rank
MISBOA	0.5269	0.5348	0.5438	0.0070	1
SBOA	0.5332	0.5367	0.5371	0.0012	3
HHO	0.5264	0.5363	0.5590	0.0091	2
PSO	0.5300	0.5410	0.5567	0.0084	5
SCSO	0.5371	0.9698	1.6232	0.3639	13
PSA	0.5273	0.5639	0.7066	0.0533	9
QIO	0.5296	0.5378	0.5565	0.0082	4
BKA	0.5258	0.5841	0.9832	0.1415	10
GJO	0.5263	0.5412	0.5546	0.0083	6
WO	0.5371	0.5531	0.6004	0.0210	8
NRBO	0.5371	0.5991	0.8486	0.1109	12
GWO	0.5305	0.5428	0.5555	0.0086	7
WOA	0.5258	0.5988	1.1248	0.1851	11

**Table 9:** The best variables of various algorithms for the planetary gear train design problem.

Algorithms	*x*_1_ (*N*_1_)	*x*_2_ (*N*_2_)	*x*_3_ (*N*_3_)	*x*_4_ (*N*_4_)	*x*_5_ (*N*_5_)	*x*_6_ (*N*_6_)	*x*_7_ (*p*)	*x*_8_ (*m*_1_)	*x*_9_ (*m*_2_)
MISBOA	37.97	26.22	22.80	24.27	23.54	87.44	3	2.00	2.00
SBOA	29.65	14.44	15.14	23.42	17.38	83.43	3	3.00	2.00
HHO	45.30	36.27	36.79	32.61	38.78	120.10	3	1.75	1.75
PSO	34.79	21.30	21.17	25.22	21.31	91.40	3	2.25	1.75
SCSO	29.13	16.91	14.33	16.51	19.09	61.85	3	1.75	1.75
PSA	41.53	30.21	23.72	23.51	13.51	86.87	3	2.00	2.25
QIO	32.24	20.60	22.03	23.53	14.39	86.52	3	2.50	2.00
BKA	34.82	25.65	25.32	23.51	21.77	87.33	3	1.75	1.75
GJO	36.84	22.21	20.38	23.60	15.43	86.79	3	2.00	1.75
WO	28.55	17.30	14.05	16.51	13.51	61.64	5	1.75	1.75
NRBO	28.02	19.14	15.93	16.51	16.92	62.24	3	1.75	1.75
GWO	52.65	22.10	14.28	23.91	19.59	86.97	3	1.75	1.75
WOA	34.52	25.60	24.57	24.28	19.87	87.06	3	1.75	1.75

**Table 10:** Results of various algorithms for the robot gripper design problem.

Algorithms	The Best	The Average	The Worst	The Std	Rank
MISBOA	2.5596	2.9459	3.3890	0.2267	1
SBOA	2.6154	3.0286	3.4375	0.3525	2
HHO	3.7227	14.7582	79.5867	23.7816	12
PSO	3.3858	4.4163	5.2854	0.6725	8
SCSO	6.8012	254.0620	1417.0476	462.3691	13
PSA	2.8181	3.7524	5.5934	0.8160	6
QIO	3.2198	3.7341	4.6100	0.4506	5
BKA	2.6099	3.3315	4.3028	0.5159	3
GJO	2.8372	3.8936	4.9890	0.6637	7
WO	3.0550	4.5285	5.9751	0.9301	9
NRBO	2.5948	5.8424	15.3426	3.6253	10
GWO	3.4269	3.7197	4.0471	0.2179	4
WOA	3.0753	6.1437	9.8143	2.0333	11

**Table 11:** The best variables of various algorithms for the robot gripper design problem.

Algorithms	*x*_1_ (*a*)	*x*_2_ (*b*)	*x*_3_ (*c*)	*x*_4_ (*e*)	*x*_5_ (*f*)	*x*_6_ (*l*)	*x*_7_ (*d*)
MISBOA	149.78	149.48	200.00	0.17	147.82	101.50	2.32
SBOA	150.00	149.84	200.00	0.00	10.10	105.01	1.60
HHO	149.99	149.17	160.93	0.04	27.52	129.18	1.77
PSO	136.95	130.70	180.44	5.97	134.17	107.89	2.42
SCSO	120.55	108.47	109.46	11.36	10.00	113.75	1.68
PSA	146.24	137.89	200.00	8.09	126.20	108.51	2.29
QIO	134.48	132.96	197.78	1.03	147.67	115.51	2.44
BKA	149.97	149.83	198.44	0.00	148.88	103.50	2.39
GJO	150.00	149.81	195.32	0.00	46.06	108.24	1.80
WO	150.00	148.54	200.00	0.50	92.26	132.93	2.12
NRBO	150.00	149.76	199.99	0.09	11.80	103.79	1.60
GWO	148.03	145.10	197.54	0.49	150.00	151.07	2.53
WOA	145.91	145.51	187.74	0.00	10.73	116.18	1.66

**Table 12:** Results of various algorithms for the four-stage gearbox design problem.

Algorithms	The Best	The Average	The Worst	The Std	Rank
MISBOA	49.80	35,629.56	137,738.51	42,104.67	1
SBOA	43.50	53,388.20	250,878.33	100,100.08	2
HHO	168,279.45	350,597.52	464,185.35	93,312.90	7
PSO	15,548.11	106,639.29	399,268.03	118,788.06	4
SCSO	567,902.76	1,171,784.84	2,793,481.23	623,190.16	13
PSA	12,274.68	162,340.57	717,276.47	213,572.86	5
QIO	46.52	60,161.92	256,594.48	98,071.12	3
BKA	9839.23	374,880.35	607,695.76	231,737.41	9
GJO	68,095.24	364,278.70	644,579.00	192,960.40	8
WO	154,445.43	403,588.62	581,976.98	156,748.27	10
NRBO	117,747.19	884,329.98	2,232,288.75	662,328.94	11
GWO	51.09	213,894.36	415,612.84	140,816.71	6
WOA	714,462.55	1,112,066.60	1,640,079.66	322,925.94	12

**Table 13:** The best variables of various algorithms for the four-stage gearbox design problem.

Algorithms	*x*_1_ (*N*_*p*1_)	*x*_2_ (*N*_*p*2_)	*x*_3_ (*N*_*p*3_)	*x*_4_ (*N*_*p*4_)	*x*_5_ (*N*_*g*1_)	*x*_6_ (*N*_*g*2_)	*x*_7_ (*N*_*g*3_)	*x*_8_ (*N*_*g*4_)
MISBOA	21	15	23	25	51	36	54	37
SBOA	20	15	20	22	43	35	40	43
HHO	10	14	40	14	43	23	41	38
PSO	25	21	23	40	56	59	50	58
SCSO	13	7	7	17	27	17	21	22
PSA	21	39	20	34	73	51	64	46
QIO	21	23	18	23	53	49	36	42
BKA	18	18	8	16	25	40	22	37
GJO	17	16	22	11	40	52	57	11
WO	7	46	12	13	25	49	30	27
NRBO	25	17	12	13	47	48	12	48
GWO	16	25	21	17	35	54	38	39
WOA	12	15	7	7	12	18	37	22
**Algorithms**	***x*_9_ (*b*_1_)**	***x*_10_ (*b*_2_)**	***x*_11_ (*b*_3_)**	***x*_12_ (*b*_4_)**	***x*_13_ (*x*_*p*__1_)**	***x*_14_ (*x*_*g*__1_)**	***x*_15_ (*x*_*g*__2_)**	***x*_16_ (*x*_*g*__3_)**
MISBOA	3.175	3.175	3.175	3.175	88.9	38.1	50.8	38.1
SBOA	3.175	3.175	3.175	3.175	38.1	50.8	63.5	38.1
HHO	3.175	3.175	3.175	3.175	25.4	50.8	50.8	38.1
PSO	3.175	3.175	3.175	3.175	25.4	76.2	76.2	50.8
SCSO	3.175	3.175	3.175	3.175	38.1	12.7	63.5	25.4
PSA	3.175	3.175	3.175	3.175	50.8	76.2	88.9	50.8
QIO	3.175	3.175	3.175	3.175	76.2	50.8	50.8	38.1
BKA	3.175	3.175	5.715	3.175	63.5	25.4	38.1	38.1
GJO	3.175	3.175	3.175	5.715	25.4	38.1	63.5	50.8
WO	5.715	3.175	3.175	3.175	50.8	88.9	50.8	25.4
NRBO	3.175	3.175	8.255	3.175	38.1	50.8	76.2	76.2
GWO	3.175	3.175	3.175	3.175	50.8	50.8	50.8	25.4
WOA	5.715	3.175	3.175	3.175	38.1	12.7	38.1	12.7
**Algorithms**	***x*_17_ (*x*_*g*__4_)**	***x*_18_ (*y*_*p*__1_)**	***x*_19_ (*y*_*g*__1_)**	***x*_20_ (*y*_*g*__2_)**	***x*_21_ (*y*_*g*__3_)**	***x*_22_ (*y*_*g*__4_)**		
MISBOA	50.8	50.8	50.8	76.2	50.8	50.8		
SBOA	38.1	101.6	63.5	63.5	63.5	50.8		
HHO	38.1	76.2	38.1	50.8	25.4	38.1		
PSO	88.9	88.9	50.8	76.2	50.8	76.2		
SCSO	12.7	12.7	12.7	12.7	38.1	25.4		
PSA	63.5	12.7	63.5	50.8	76.2	63.5		
QIO	50.8	25.4	63.5	76.2	38.1	50.8		
BKA	38.1	25.4	38.1	63.5	63.5	63.5		
GJO	50.8	12.7	50.8	50.8	50.8	12.7		
WO	25.4	101.6	76.2	38.1	76.2	76.2		
NRBO	76.2	63.5	25.4	38.1	38.1	76.2		
GWO	50.8	25.4	76.2	76.2	50.8	76.2		
WOA	63.5	38.1	38.1	12.7	12.7	25.4		

**Table 14:** The results of various algorithms for TSP.

Algorithms	The Best	The Average	The Worst	The Std	Rank
MISBOA	7.3535	7.4945	7.6473	0.0829	1
SBOA	7.3659	7.5073	7.6431	0.0941	2
HHO	7.7392	8.2628	8.9568	0.3604	11
PSO	7.6862	7.9092	8.1677	0.1372	8
SCSO	8.1210	8.5124	8.9007	0.2366	13
PSA	7.5284	7.8043	8.2786	0.2163	7
QIO	7.5307	7.6551	7.7299	0.0653	3
BKA	7.5619	7.7585	8.0241	0.1609	5
GJO	7.8943	8.2646	8.5822	0.2020	12
WO	7.5249	7.7156	7.8352	0.1005	4
NRBO	7.6937	7.9233	8.2097	0.1570	9
GWO	7.6019	8.0283	8.6042	0.3257	10
WOA	7.5243	7.7743	7.9464	0.1348	6

**Table 15:** The results of various algorithms for shape optimization problems of combined curves.

Algorithms	The Best	The Average	The Worst	The Std	Rank
MISBOA	25,999.74440	25,999.74440	25,999.74442	5.3021E-06	1
SBOA	25,999.76483	26,000.06963	26,000.85249	3.6657E-01	5
HHO	25,999.74440	25,999.74494	25,999.74760	1.0275E-03	2
PSO	26,025.83962	26,044.24748	26,074.01958	1.2059E+01	13
SCSO	25,999.74463	25,999.78421	25,999.94426	6.3569E-02	4
PSA	26,018.82949	26,028.23579	26,040.48786	7.5703E+00	12
QIO	25,999.74440	26,000.12618	26,001.97965	6.8187E-01	7
BKA	25,999.74534	26,001.61971	26,004.55399	1.9536E+00	8
GJO	26,003.03461	26,009.58403	26,016.95296	5.5396E+00	10
WO	25,999.74441	26,000.11604	26,001.53414	7.1334E-01	6
NRBO	25,999.74441	25,999.76224	25,999.87262	4.0222E-02	3
GWO	26,016.19124	26,026.47146	26,061.95977	1.3641E+01	11
WOA	25,999.76399	26,006.30961	26,018.65573	6.5048E+00	9

## Data Availability

All data generated or analyzed during the study are included in this published article.
